# The Critical Criterion on Runaway Shear Banding in Metallic Glasses

**DOI:** 10.1038/srep21388

**Published:** 2016-02-19

**Authors:** B. A. Sun, Y. Yang, W. H. Wang, C. T. Liu

**Affiliations:** 1 Centre for Advanced Structural Materials, Department of Mechanical and Biomedical Engineering, City University of Hong Kong, Kowloon, Hong Kong; 2 Institute of Physics, Chinese Academy of Sciences, Beijing, 100190, China

## Abstract

The plastic flow of metallic glasses (MGs) in bulk is mediated by nanoscale shear bands, which is known to proceed in a stick-slip manner until reaching a transition state causing catastrophic failures. Such a slip-to-failure transition controls the plasticity of MGs and resembles many important phenomena in natural science and engineering, such as friction, lubrication and earthquake, therefore has attracted tremendous research interest over past decades. However, despite the fundamental and practical importance, the physical origin of this slip-to-failure transition is still poorly understood. By tracking the behavior of a single shear band, here we discover that the final fracture of various MGs during compression is triggered as the velocity of the dominant shear band rises to a critical value, the magnitude of which is independent of alloy composition, sample size, strain rate and testing frame stiffness. The critical shear band velocity is rationalized with the continuum theory of liquid instability, physically originating from a shear-induced cavitation process inside the shear band. Our current finding sheds a quantitative insight into deformation and fracture in disordered solids and, more importantly, is useful to the design of plastic/tough MG-based materials and structures.

Shear banding across different length scales has been a principal deformation mode that accommodates plastic flow in a variety of disorder systems, from atomic thin films^1^ and amorphous solids^2^,^3^ to geologic earth faults^4^,^5^. Under compressive stress, a shear band often proceeds in a stick-slip fashion and ultimately transits into a runaway “defect”, resulting in a large catastrophic event and material failure^6^. Identifying the key factor controlling such a slip-to-failure transition is critical, which can find many important applications in natural science and engineering, such as industrial friction and lubrication^1^, material deformation and fracture^7^ and earthquakes^8^, to name but a few. This is particularly so for metallic glasses (MGs)^9^, which are a new class of amorphous material with superb strength but poor ductility due to the formation of highly localized nanoscale shear bands^10^,^11^,^12^. Although MGs possess many attractive and unique properties, the lack of a thorough understanding of the shear-band instability process, which underpins the quasi-brittle fracture behavior of MGs, however impedes their wide applications in various fields as a structural material^2^,^13^. On the other hand, with a relatively simple atomic structure and remarkable tuning properties^14^, MGs also sever as ideal model systems to investigate the fundamental issues above.

Generally speaking, once formed, a shear band can quickly turn into a runaway defect under tension, resulting in almost zero macroscopic ductility, or propagate in a stick-slip manner under compression, leading to some plastic strain even though there is only one shear band operative before final failure. Consequently, there is a general correlation between shear-band stability and plasticity in MGs. However, previous extensive studies^2^,^15^ also showed that the shear-banding mediated failure process is rather complicated, which is dependent on not only some intrinsic factors, such as alloy composition and the internal energy state of a glassy structure^15^,^16^, but also various extrinsic factors, such as sample size^17^, testing strain rate^18^, testing temperature and hydrostatic pressure^18^,^19^ as well as the stiffness of testing machine^17^. Arising from these shear complexities, the issues that have been puzzling us are: why could a single shear band sustain different plasticity? And what are key factors controlling the shear-band instability and thus the shear catastrophic failure of MGs under various conditions?

To elucidate the confounding effects of these factors, a number of phenomenological criteria were proposed in past decades. One well-known example is the Poisson’s ratio criterion proposed by Lewandowski *et al*.^15^, according to whom a MG is expected to behave in a brittle manner for its Poisson’s ratio below the critical value of ~0.32 or in a ductile manner for above^20^. Additionally, based on the energy balance principle and shear-band dynamics, recent studies^17^ also indicated that the confounding effect of sample size and machine stiffness on shear-band stability can be captured with a sole parameter, namely, the so-called shear-band instability index^17^,^21^. Despite all these prior efforts, however, a quantitative understanding of the physical process that leads to shear-band failure is still lacking. Here, through extensive experimental efforts combined with theoretical modeling, we would like to show that a shear band becomes unstable in MGs once its velocity reaches a critical value, regardless of the various intrinsic and extrinsic factors involved throughout the shear-banding process. Furthermore, we can also show that the emergence of such a critical shear band velocity is rooted into a process of liquid-type instability, which sheds important insights into the nature of shear banding mediated failure in disordered solids and helps understanding the strategies that enables tuning plasticity/ductility in MG-based materials and structures.

## Stick-slip dynamics and instantaneous shear-band velocity

**Figure 1:**
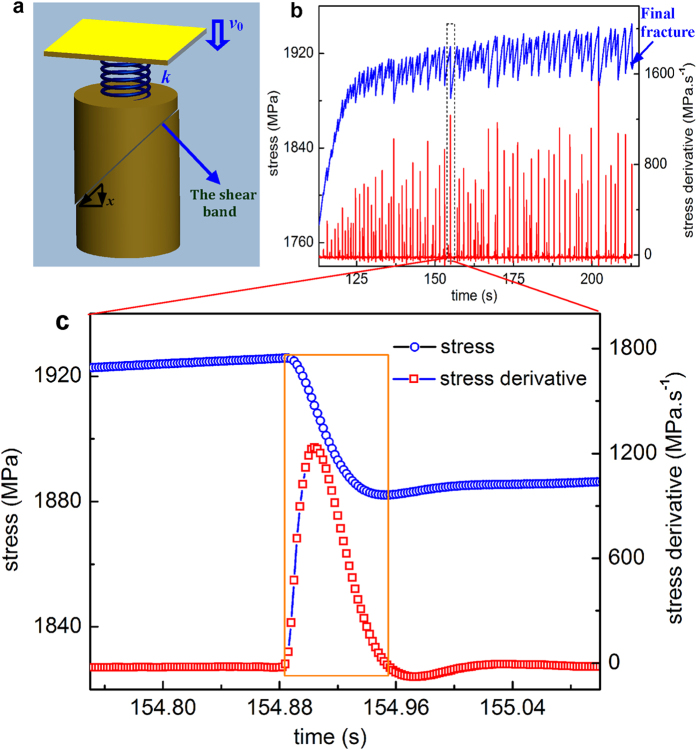
(**a**) The schematic diagram of the machine-sample system in the compression under the constant loading rate 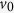. The shear band sliding will cause the release of the elastic energy and the stress drop. (**b**). Typical stress-time 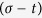 and stress derivation-time curve 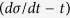 for a Vit105 sample with 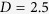 mm under the constant strain rate 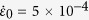 s^−1^ and the testing machine with a stiffness 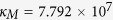 N.m^−1^. (**c**). An enlarged view for a stress serration and the stress derivative spike taken from (**b**), from which one can see that the variation of both *σ* and 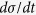 in a shear banding event can be well resolved with the current sampling rate.

As already proved by many previous studies^21^,^22^, the serrated or pop-in flows on stress-strain/time curves of monolithic bulk MGs are mainly caused by the intermittent sliding or the stick-slip process of a single dominant shear band along the shear plane, especially in the final stage of plastic regime. The shearing process on the plane was shown to be simultaneous, in contrast to the progressive shear banding across the sample before the yielding point in recent studies^23^. Based on this understanding, the shear band velocity (SBV) or the sliding velocity of materials along the shear plane can be measured using the displacement burst and the elapse time of a serration event. Usually, to obtain an accurate measurement of SBV, strain gauges with a high temporal and spatial resolution have to be used and the position of a shear band needs to be located in advance^24^,^25^. Here, we take a totally different approach to measure the SBV by exploiting the stick-slip dynamics of a single shear band. As shown in Fig. 1a, the operation of the shear band will cause the release of the elastic energy stored in the machine-sample system, thus partially relaxing the compressive load. Assuming the loading, *P*, started at the time *t* = 0, the applied stress 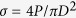 on a cylindrical sample of diameter *D* at the time *t* can be expressed as^18^,^26^:


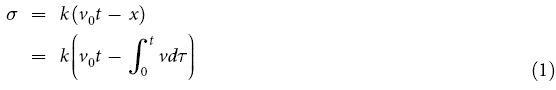


where *v*_*0*_ is the loading velocity; *x* is the vertical displacement caused by the shear band operation; *v* is the instantaneous SBV and *k* is the elastic constant of the machine-sample system, which is expressed as 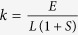 for a cylindrical sample^26^, where *E* and *L* are sample Young’s modulus and length, respectively; *S* is the ratio of sample stiffness *κ*_*S*_ to machine stiffness *κ*_*M*_: 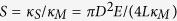. By differentiating Eq. 1 with respect to time *t*, we obtain the instantaneous SBV 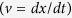 as:


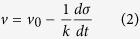


From Eq. 2, one can infer that the SBV profile with time for a shear band can be measured if the derivative of stress versus time curve 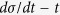 could be experimentally resolved. It should be noted that the spatial properties of the shear band are average out in the derivation of Eq. 2. This is reasonable for a mature shear band in the later stage of plastic deformation close to the fracture.

## Measurement of Shear Band Velocity

**Figure 2:**
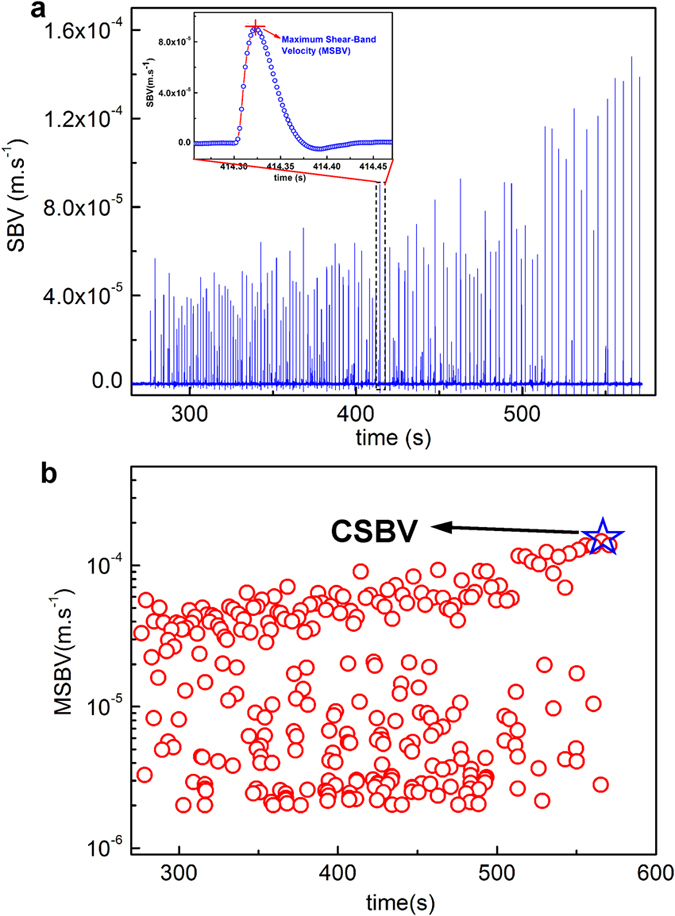
(**a**) A typical shear-band velocity (SBV) profile 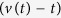 in the whole plastic deformation regime before final fracture for a sample (Vit105, *D* = 2 mm, 
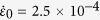
 s^−1^ and 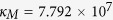 N.m^−1^). One can see that each shear-band slipping event corresponds to a velocity burst in the profile, from which the maximum shear-band velocity (MSBV) can be determined, as shown in the inset. (**b**) The distribution of extracted MSBV with time for the SBV profile in (**a**), where one can obtain a maximum value among all MSBVs and the value is defined as the critical shear-band velocity (CSBV) for the instability.

To measure the SBV, compressive stress-time curves of MGs under various testing conditions (90 specimens in total, as listed in Table S1 in Supplementary Information(SI)) until the final catastrophic failure were obtained with a data-sampling rate of 500 Hz. All curves were subsequently differentiated with time to yield the stress derivatives. As shown in Fig. S1 in SI, the stress signals acquired has a noise level of <0.8 MPa and show relatively smooth and continuous stress profile than those obtained with a piezoelectric load cell^27^,^28^, thus facilitating the subsequent differential analysis. Figure 1b displays the typical stress-time (*σ* − *t)* and stress derivative-time (*dσ/dt* − *t*) curves for a Vit105 sample with *D* = 2.5 mm under the constant strain rate 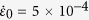 s^−1^. As seen from these two curves, each serration event on the *σ* − *t* curve corresponds to a spike on the *dσ/dt* − *t* curve, indicating a sudden stress drop triggered by the shear band operation. The stress drop is well resolved with the current data sampling rate, as shown in the enlarged view in Fig. 1c. With the curve of stress derivative versus time being well captured, the shear band velocity profile can be calculated for various MG samples according to Eq. 2. The calculated values of *k* (*k* = *E*/[*L*(1 + *S*)]) and *v*_*0*_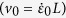 for each MG sample are listed in Table S1. To calculate the parameter, *k*, values of different testing machine stiffness, *κ*_*M*_ , are experimentally determined (see Fig. S2 in SI). Figure 2 shows a typical shear band velocity profile (*v*(*t*) − *t*) (Vit105, *D* = 2 mm, 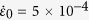 s^−1^ and 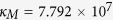 N.m^−1^), from which one can clearly see that, at the very beginning of the slip event, the shear band velocity quickly rises to a maximum, and then rapidly drops to zero. A similar process occurs until the next slip event is triggered, conforming to a typical stick-slip dynamic behavior. For each serration or slip event, we can extract a maximum shear-band velocity (MSBV), located at the inflection point of the stress-time curve. In principle, we can extract many MSBVs from a single stress-time curve, as exemplified in Fig. 2b, which displays the distribution of extracted MSBVs over the testing time of a single experiment. The environmental noise also causes tiny spikes on the *dσ/dt* − *t* curve; however, the magnitude of these tiny spikes is very small and can be estimated to be less than 2 × 10^−6^ m.s^−1^ (see Fig. S3 in SI). Therefore, to remove the noise effect, spikes with a magnitude less than 2 × 10^−6^ m.s^−1^ are discarded from the MSBV analysis.

**Figure 3:**
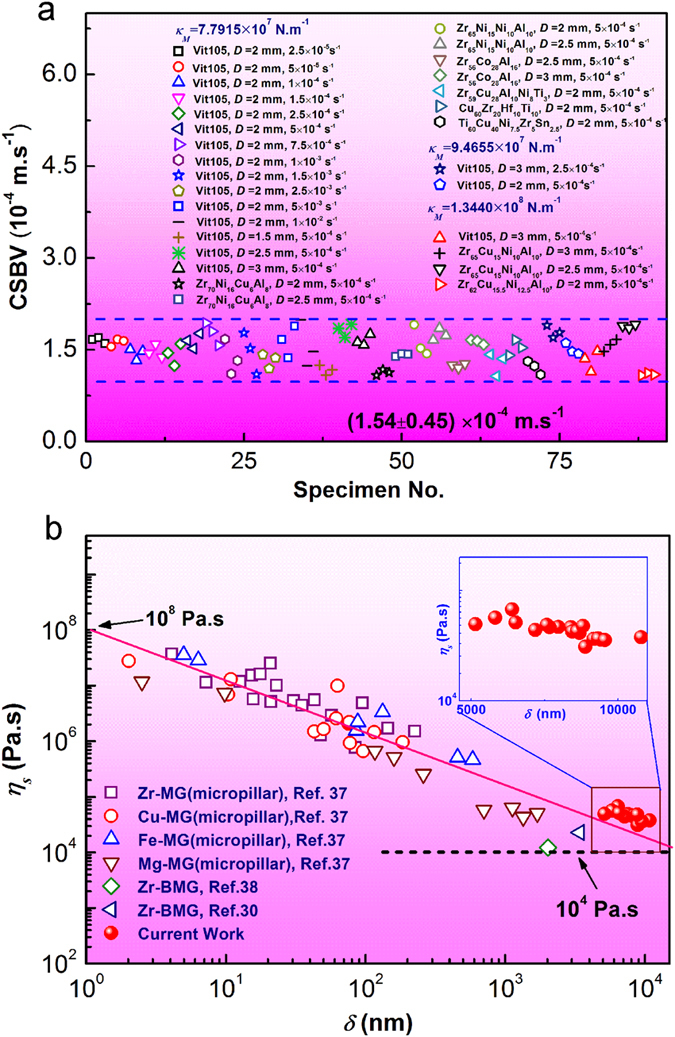
(**a**) The measured values of CSBV for MG samples under various intrinsic and extrinsic conditions, from which one can see that these values are concentrated on a narrow range 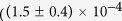 m.s^−1^). (**b**) The shear-band viscosity (*η*_*S*_) collected from MG literature and our current work, which are plotted with the shear offset (*δ*) which scales with the sample size. One can see at the high end, these values approach a lower viscosity limit (10^4^ Pa.s) for shear-band instability.

As the serrated plastic flow continues, the shear band finally becomes unstable and develops into a crack, leading to the catastrophic shear failure of MGs. In general, it is difficult to pinpoint the critical shear band velocity (CSBV) right at the moment of crack formation due to the short time duration of the final fracture process. The typical stress-time profile for the final fracture event is shown in Fig. S4 in SI for the comparison with those of the serrated events. Due to the interruption of crack formation and propagation, the stress dropped much rapidly, thus generating a pronounced spike in comparison to the serrated events before the fracture. Alternatively, we chose to pick out the largest MSBV among all those detectable MSBVs. As this is the largest velocity that the band could experience before the final fracture process, it is reasonable to take it as an approximation of the CSBV for a MG sample. In doing so, we obtain the CSBVs for all MG samples, as listed in Table S1 and shown in Fig. 3a.

## Constant CSBV Governing Slip-to-Failure Transition

From Fig. 3a, it is surprising to see that the CSBV for all MG samples are around a constant value, i.e., (1.5 ± 0.4)×10^−4^ m.s^−1^, regardless of alloy compositions, sample sizes, strain rates and testing machines. One should note that the CSBV values measured with our methods is much smaller than those measured with the acoustic emission technique^29^. The reason may be that shear banding is a dynamic process that involves multiple stages of plastic deformation, including the nucleation of a shear “embryo” and the simultaneous sliding along a fully-grown shear plane. In principle, the nucleation stage entails progressive shearing and the shearing speed of the front could approach the sound speed; in contrast, the sliding stage is a stick-slip process where the shear speed is slow which depends on various extrinsic and intrinsic factors. In principle, it can be anticipated that the acoustic emission results should correspond to the first shearing speed, as associated with the propagation of a stress wave from the shearing front (progressive shearing).

As all MG samples in our work are mainly deformed by the formation of a single shear band (see Fig. S5 in SI), this constant CSBV indicates that the single shear-band stability under various experimental conditions is governed by a critical velocity *v*_*c*_ above which the shear banding in MGs transits from the stick-slip sliding to the catastrophic failure. A recent work by Wu *et al*.^30^ showed that the runaway shear band instability also correlates with a critical shear energy density, which is dependent on the glassy composition but independent of sample sizes. Since both the CSBV reported here and the critical shear energy density stem from the stick-slip shear banding process, one might expect some correlation between them. As the composition effect is mainly reflected by the factor *k* or the modulus *E*, it is sensible that chemical composition should play a role in shear banding, at least in the stable shear-banding regime. The insensitiveness of the CSBV to the chemical composition has an important physical implication. As will be discussed later, the CSBV in fact reflects the liquid-type instability of shear banding, which is governed by a critical viscosity and constant shear band thickness independent of the compositions.

If one simply assumes that the shear-band thickness λ, is a constant (e.g. ~10 nm based on the observation of electronic microscopy^31^, the constant CSBV in fact implies a critical shear strain rate, 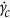, which is estimated to be on the order of 10^4^ s^−1^, according to the equation, 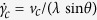, with *θ* the shear angle of the band. This strain rate is almost 9 orders of magnitude larger than the applied strain rate (10^−4^ ~ 10^−3^ s^−1^). As discussed below, some dynamic instability processes would occur to the sheared glassy materials under such a high strain rate, triggering the crack formation and propagation and thus the catastrophic failure.

Aside from the critical shear strain rate, we can also obtain the critical viscosity for all MG samples at which the shear band is about to become unstable, from the shear stress (*τ* = σ/2)^32^ and the shear strain rate corresponding to the CSBV, which are summarized in Fig. 3b and Table S1. From these results, one can see that the values of the critical viscosity at the onset of catastrophic failure are concentrated in a narrow range around 10^4^ Pa.s, a typical viscosity value of supercooled liquids well above the glass transition temperature. For comparison, we also collected from literature^34^ the reported shear-band viscosity values measured from MGs of various sizes, ranging from the μm-scale to the mm-scale. These values are plotted against the shear offset, *δ*, caused by the individual shear band. In our work, the shear offset value is indirectly obtained from the stress drop, Δσ_s_, in a serration event according to the equation, *δ* = (*v*_0_Δ*t*_*s*_ + Δσ_s_/*k*) (see the detailed derivation in SI), where Δ*t*_*s*_ is the duration of the serrated event. From Fig. 3b, one can see a clear negative correlation between the shear-band viscosity and the shear offset. As the shear offset of a shear band is scaled with the sample size^21^, the general trend seen between the shear band viscosity and the shear offset in Fig. 3b implies a size-dependent shear band viscosity. What is really worth noting here is the viscosity values at two ends of the trend, being respectively relevant to the shear-band initiation and instability processes. On the end where *δ* is extrapolated to zero, the *η*_*s*_ exhibits a value of 10^8^ Pa.s, which is very close to the viscosity of “flow units” or liquid-like sites that can be extracted experimentally through inelasticity in MGs^33^,^35^,^36^. This behavior suggested that the shear band initiation is rooted in the activation of the liquid-like sites or “flow units”, as consistent with the recent findings reported in ref. 33. On the other end, one can see that *η*_*s*_ is approaching a plateau value of 10^4^ Pa.s^−1^, seemingly independent of *δ* when its magnitude varies in the micrometer range. As seen in the later text, this viscosity limit at which a shear band becomes unstable provides the quantitative evidence to unravel the physical origin of the shear band stability in MGs.

## Discussion

First, it is worth pointing out that the time duration of the serrated event (~80 ms) measured with our experimental setup is much larger than those (~1–10 ms) reported in the literature. There are two possible reasons for this discrepancy: (i) the MG composition and size as well as the testing rate and temperature in our tests are different from the previous studies^25^,^26^,^27^,^28^. These factors are well known to affect the serration events, such as their size and time duration^18^,^28^; (ii) we used a commercial load cell with a lower low-pass filter which has the bandwidth of 100 Hz. This does not affect the load drop measurement but may “flatten” a sharp serration event during the data recording. However, the SBV measured here refers to an instantaneous speed, which is different from the average SBV reported previously^25^,^26^,^27^,^28^. In principle, the value of the average SBV depends largely on the precise determination of the time duration while that of the instantaneous SBV mainly depends on the slope of the stress-time profile, which weakly depends on the time duration of a serration event. This may explain that, even though the time duration we measured is almost two orders of magnitude larger than those reported previously, the SBV value we obtained is in the same order of magnitude with those (10^−4^–10^−3^ ms^−1^) already reported^25^,^26^,^27^,^28^. In this work, our intention is to find a critical criterion for the instability of the shear banding process under different testing conditions; therefore, it is meaningful to compare the SBV values obtained with the same experimental technique under different testing conditions.

**Figure 4:**
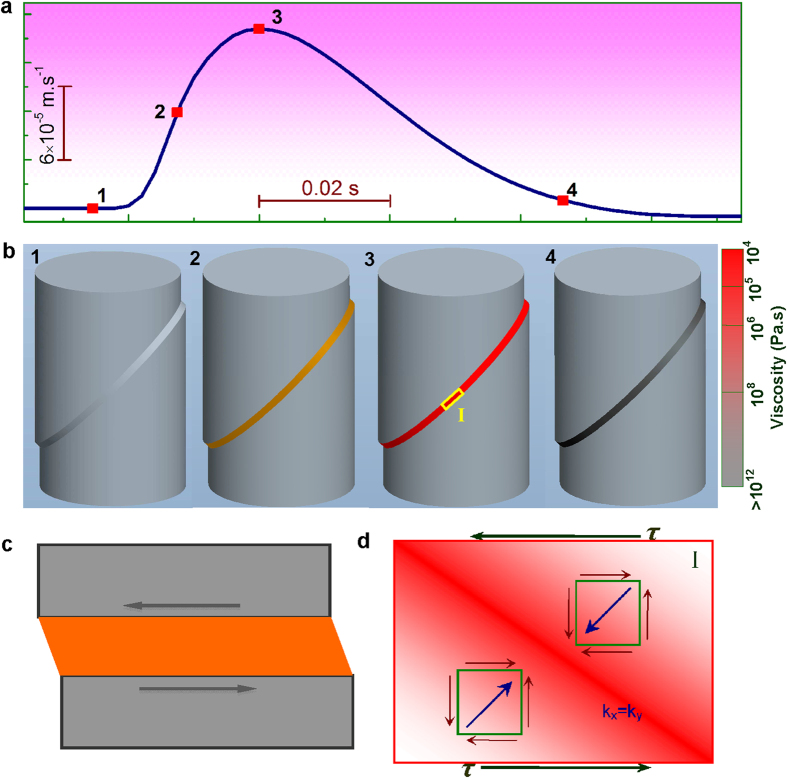
(**a**) The variation of SBV in a critical serrated event. (**b**) The schematic of the viscosity change in the band at different stages of shear band sliding. (**c**) The schematic for a shear band slipping which can be viewed as a viscous liquid layer sheared between two plates moving relatively with each other. (**d**) The schematic for the cavitation instability due to coupling between the density fluctuation and stress deformation field inside the band. The evolution of the thermal density fluctuation mode 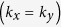 under shear stress as indicated by the black arrows and the sum of these viscous stress acting on each region is shown by thick red arrows.

To understand the emergence of CSBV, let us turn back to the physical origin of shear banding, which is related with the strain softening in MGs. While it is still controversial on the physical origin of strain softening, i.e. whether it is caused by stress-induced structure disordering or temperature rise^37^,^38^, there were unambiguous experimental evidence^34^,^39^ reported for the significant softening within a living shear band, which showed an apparent viscosity close to that of glass transition or well above. Thus, a sliding shear band can be reasonably treated as a viscous liquid layer under a simple shear stress, as schematically shown in Fig. 4. Based on this view, the shear-band instability, which leads to the final fracture of MGs, can be well analyzed within the framework of fluid dynamic instability. In principle, a liquid layer subject to a shear stress could turn into a runaway defect because of either temperature rise^40^ or excessive straining^41^, both of which could be related to a critical velocity. However, by viewing the shear band as a two-dimensional heat source with a heat flux scaling with the band velocity and the sample body as a three-dimensional heat-conducting medium^21^, we calculated the instantaneous temperature rise Δ*T* during a serration event based on the measured shear band velocity profile (See details in SI). The numerical integration calculation showed that the maximum temperature rise Δ*T*_*max*_ during the slip event is less than 5 K. Therefore, it is unlikely that the slip-to-failure transition would be caused by a temperature surge within the shear band. Nevertheless, this does not exclude the scenario that the temperature within a shear band can rise up to a very high value at the later stage of crack propagation when the velocity of the advancing crack tip reach a high value, as already discussed in refs 46 and 47. Now, let us focus on the possible mechanisms for the slip-to-failure transition for the shear band remaining “cold” during an isothermal process.

Without taking into account the temperature change, the shear-band instability can still be triggered either from outside of the band or from inside. The former is often associated with fluid meniscus instability, which has been extensively discussed in the fracture literature of MGs^7^,^42^. However, a typical process of the meniscus instability is often associated with a mode I fracture in a liquid layer with a finite thickness^42^, which is contrasting the shear band movement in a compression test, in which shear banding only causes relative shearing of materials along the thin liquid layer. Therefore, the mechanism of meniscus instability is unlikely to govern the shear instability in compression testing. On the other hand, according to the recent theory of Furukawa and Tanaka^43^, only simply shear stress alone can cause instability within a liquid whose local density is intrinsically heterogeneous or fluctuating. This process occurs as the shear-induced enhancement of density fluctuation is self-amplified by the enhancement of dynamic and elastic asymmetry between denser and less dense regions, and results in cavitation in shear band, which has been verified both by experiments and theoretical studies^44^,^45^,^46^. By incorporating the general viscoelastic constitutive equations and free-volume concept of glassy materials into the Navier-Stokes equation, Furukawa and Tanaka quantitatively showed that, in theory, a compressible viscoselastic glassy liquid can become mechanically unstable under the simple shear above a critical shear strain rate 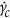:


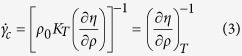


where *ρ* and *ρ*_*0*_ are the mass density and the average mass density, respectively. *K*_*T*_ is the isothermal compressibility, *η* and *p* are the viscosity and the pressure of the liquid, respectively. The shear instability occurs for 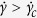 and 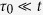 (*τ*_0_ and *t* are the characteristic structural relaxation time of the liquid and the experimental characteristic time, respectively). As an operating shear band can be reasonably viewed as a viscous glassy liquid layer under the simple shear (See Fig. 4c), it can be readily obtained from Eq. 3 that the shear-band instability is controlled by a critical strain rate or velocity if one assumes the shear-band thickness is a constant. Taking the shear band thickness ~10 nm, we obtain a critical strain rate on the order of 10^4^ s^−1^ from the measured CSBVs or the magnitude of 
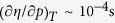
 at the critical point of shear instability. In contrast to the 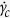 of 10^−5^ ~ 10^0^ s^−1^ obtained above the glass transition point from the macroscopic Vitreloy 1 sample^38^, the 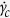 with a value as high as 10^4^ s^−1^ measured from a shear band suggests that the viscous glassy material in the shear band has a much larger failure resistance than the normal glassy materials in the supercooled liquid regime. Furthermore, it is worthy to mention that the pressure dependent shear instability may be related with the pressure dependence of plastic flow and fracture behavior of the MGs, as reported in many previous studies^19^,^47^,^48^, the details of which deserves further research.

**Figure 5:**
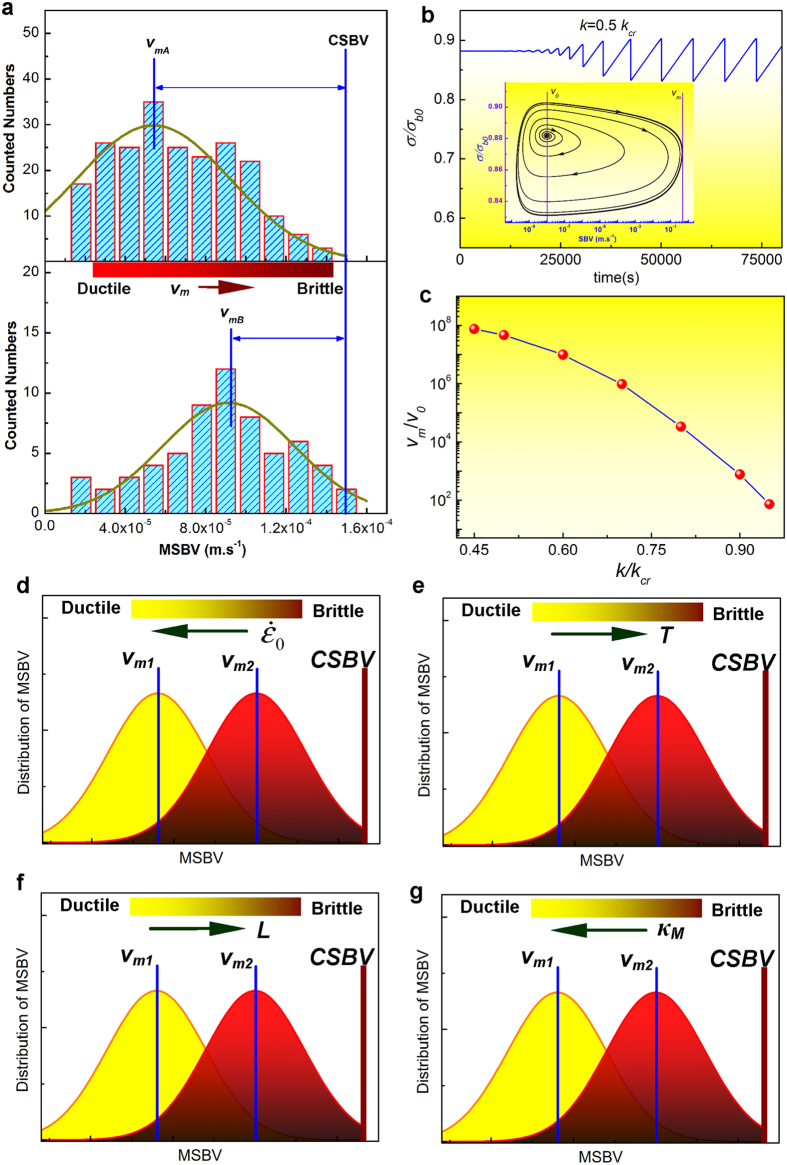
(**a**) The number distribution of MSBV versus MSBV for two typical MG samples with different ductility level: Zr_70_Ni_16_Cu_6_Al_8_ 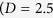 mm) (the top) and Zr_65_Cu_15_Ni_10_Al_10_ (*D* = 2 mm) (the bottom) both under 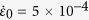 s^−1^ and 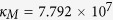 N.m^−1^. Both histograms can be fitted by the Gauss distribution with a mean *v*_*m*_ and terminate at the same CSBV. (**b**) A typical numerically calculated stress-time curve for 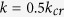, the inset shows the stress-SBV phase diagram where the initial perturbation on the steady sliding of shear band gradually develops into stable stick-slip cycles with a characteristic maximum velocity 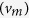. (**c**) The calculated variation of *v*_*m*_ with 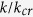. (**d**–**g**). Schematic illustrations of the variation *v*_*m*_ with different factors (the strain rate 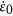, the testing temperature *T*, the sample size *L* and the testing machine stiffness *κ*_*M*_), thus yielding different ductility of MGs.

Since the CSBV governing the shear-band instability is a constant, tuning the shear-band stability for enhancing the ductility of MGs is possible by means in favor of the slow-down of the SBV, such as optimization of elastic moduli, sample size reduction, testing strain rate and temperature adjustment, etc. Figure 5 displays the typical histograms of the MSBVs counted over the entire plastic deformation regime for two MG alloys. It is evident that both histograms exhibit a single peak and can be fitted to a Gaussian distribution but with different mean values, *v*_*mA*_ and *v*_*mB*_ (*v*_*mA*_ < *v*_*mB*_). Despite that, both histograms terminate at the same CSBV due to the shear-band instability. Since the larger is the characteristic shear band velocity *v*_*m*_, the higher is the probability that the shear band could reach the CSBV given the same velocity fluctuations around *v*_*m*_, it can be thus inferred that the sample with a larger characteristic shear band velocity is more prone to catastrophic shear failure and, therefore, displays lower shear stability or ductility. Indeed, this argument can be verified from our current work that the MG with *v*_*mA*_ has a much larger plasticity (the fracture strain *ε*_*p*_ ~ 11.2%) than that with *v*_*mB*_ (*ε*_*p*_ ~ 5.4%). Furthermore, from the theoretical stability analysis of the stick-slip dynamics along a single shear band^18^ (also see the numerical calculations in SI), we can show that the *v*_*m*_ of a single shear band under various conditions is negatively correlated with the ratio of *k/k*_*cr*_, as shown in Fig. 5b,c, where *k* is a function of sample modulus, sample size as well as the machines stiffness (e.g. *k* *=* *E/*[*L* + (*πD*^2^*E*/4*κ*_*M*_)] for a cylinder sample), while *k*_*cr*_ is a function of the testing strain rate and temperature. Specifically, *k* increases with the reduced sample size or the increased *κ*_*M*_, while *k*_*cr*_ decreases with the increased loading strain rate or the decreased temperature. In such a case, a smaller sample size, a larger testing frame stiffness, a higher strain rate and lower testing temperature results in a higher value of *k*/*k*_*cr*_ and thus a lower value of *v*_*m*_ which favors ductility in MGs (see Fig. 5d–g) given the plastic flow being still governed by the stick-slip dynamics 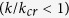. These predictions are in general accordance with previous experimental findings^17^,^28^. Furthermore, it is worth mentioning that *k* decreases monotonically with the increasing Young’s modulus *E* and *k*_*cr*_ is also a function of the shear modulus *G* (see SI), therefore the ratio of *k*/*k*_*cr*_ is a complex function of the Poisson’s ratio *v*. This suggests that the plasticity in MGs should be intrinsically correlated with their Poisson’s ratio, as originally proposed in ref. 14, even from the perspective of stick-slip shear-banding dynamics. With the above knowledge, one can adjust the characteristic velocity of a shear band by varying the various factors, extrinsic or intrinsic, and tune the shear-band stability or ductility of MGs. In principle, by keeping *v*_*m*_ away from the CSBV, we could obtain a highly stable shear band and thus a ductile MG.

Finally, it is worth noting that the constant CSBV for shear-band instability as found in our present work is also helpful to understanding the dynamics of diverse systems across different length scales^1^,^4^,^5^, e.g. peeling off of an adhesive tape, the friction and lubrication, seismic and geodetic fault slip, etc. Despite the rather different physical nature of these problems, the dynamic systems involved undergo a similar process of shear localization, from stick-slip sliding to the occurrence of disastrous events (e.g. earthquakes or landslides). In view of the commonality of the phenomena that one can perceive between the general nonlinear dynamics and shear banding in MGs, the finding of the constant CSBV for the MGs selected for this study may provide a quantitative insight into the failure mechanisms of the dynamic systems involving liquid-like phases, which one may encounter in the broad discipline of natural science and engineering.

## Methods

MG alloy ingots with the nominal composition listed in Table S1 were produced by arc melting a mixture of pure metals (purity 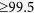
% in mass weight) in a Ti-gettered argon atmosphere. To ensure the compositional homogeneity, each ingot was remelted at least four times. Rod-shape MG samples with a set of different diameters (1.5 mm, 2 mm, 2.5 mm and 3 mm) and a length of at least 30 mm were obtained by suction casting into a copper mould. The amorphous nature of the as-cast specimens was confirmed by the x-ray diffraction XRD, PANalytical X’Pert PRO) with Co Ka radiation and the differential scanning calorimetry (DSC, Perkin Elmer DSC7). Samples with different diameters were cut from MG rods by a diamond saw with water cooling, and then carefully ground into compression specimens with an aspect ratio of 2:1 within an accuracy of ±5 μm at two ends. The room-temperature uniaxial compression tests were mainly performed on an Instron 5567 electromechanical test system under the displacement-controlled mode. The sample composition, size and the testing strain rate are varied to examine the effect of these factors on the shear band velocity. To examine the influence of the testing machine, some samples were also tested on the Instron electromechanical test system 3384 and 5869, respectively. In order to get the machine stiffness, all testing machines are also loaded without sample at different displacement rates. For all tests, the load, the displacement and the time are recorded at a high frequency 500 Hz. Each test at the same condition is performed at least 3 times, to obtain the reliability of the data. After the test, the shear band and fracture surface morphology are investigated by a scanning electron microscopy (SEM, Gemini1530).

## Additional Information

**How to cite this article**: Sun, B. A. *et al*. The Critical Criterion on Runaway Shear Banding in Metallic Glasses. *Sci. Rep*. **6**, 21388; doi: 10.1038/srep21388 (2016).

## Supplementary Material

Supplementary Information
