# Supplementary material for: The Critical Criterion on Runaway Shear Banding in Metallic Glasses
Source: Sci Rep. 2016 Feb 19;6:21388. doi: 10.1038/srep21388 (PMC4759565; doi:10.1038/srep21388)
Supplement: Supplementary Information [file srep21388-s1.doc]

**Supplementary Information for “The Critical Criterion on Runaway Shear Banding in Metallic Glasses”**

B.A. Sun^1^, Y. Yang^1,*^, W. H. Wang^2^, C. T. Liu^1,*^

^1^Centre for Advanced Structural Materials, Department of Mechanical and Biomedical Engineering, City University of Hong Kong, Kowloon, Hong Kong

^2^Institute of Physics, Chinese Academy of Sciences, Beijing, 100190, China

**Correspondence and requests for materials should be addressed to C. T. L (**[**chainliu@cityu.edu.hk**](mailto:chainliu@cityu.edu.hk)**) and Y. Y (yonyang@cityu.edu.hk).**

**Supplemental materials including**

**Table SI**

**Text S1-S3**

**Fig. S1-S6**

**References**

**Table S1**. The MG compositions, the Young’s Modulus *E,* sizes (the diameter, *D*, and the height, *L*), the testing strain rates , the calculated *k* ($k=E/[L(1+S)]$), the measured critical velocity for shear-band instability $v_{c}$ with the mean $\bar{v}_{c}$ and error range $err(v_{c})$ for each sample tested at the same condition.

| **Compositions** | *E*  **(GPa)** | *D*  **(mm)** | *L*  **(mm)** | **(s^-1^)** | *k*  **(GPa.m^-1^)** | *v_c_*  **(10^4^m.s^-1^)** | **Specimen**  **No.** | $\bar{v}_{c}$ **(10^4^m.s^-1^)** | $err(v_{c})$ **(10^4^m.s^-1^)** |
| --- | --- | --- | --- | --- | --- | --- | --- | --- | --- |
| *Instron 5567*, $\kappa_{M}=7.79\times10^{7}$N.m^-1^ | | | | | | | | | |
| Zr_52.5_Ti_5_Cu_17.9_Ni_14.6_Al_10_ (Vit105) | 88 | 2 | 4 | 2.5×10^-5^ | 11657 | 1.67 | 1 | 1.66 |  |
|  |  |  |  |  |  | 1.70 | 2 |  |  |
|  |  |  |  |  |  | 1.60 | 3 |  |  |
| Vit105 | 88 | 2 | 4 | 5×10^-5^ | 11657 | 1.55 | 4 | 1.62 |  |
|  |  |  |  |  |  | 1.67 | 5 |  |  |
|  |  |  |  |  |  | 1.64 | 6 |  |  |
| Vit105 | 88 | 2 | 4 | 1×10^-4^ | 11657 | 1.51 | 7 | 1.44 |  |
|  |  |  |  |  |  | 1.33 | 8 |  |  |
|  |  |  |  |  |  | 1.47 | 9 |  |  |
| Vit105 | 88 | 2 | 4 | 1.5×10^-4^ | 11657 | 1.44 | 10 | 1.47 |  |
|  |  |  |  |  |  | 1.59 | 11 |  |  |
|  |  |  |  |  |  | 1.38 | 12 |  |  |
| Vit105 | 88 | 2 | 4 | 2.5×10^-4^ | 11657 | 1.45 | 13 | 1.43 |  |
|  |  |  |  |  |  | 1.24 | 14 |  |  |
|  |  |  |  |  |  | 1.60 | 15 |  |  |
| Vit105 | 88 | 2 | 4 | 5×10^-4^ | 11657 | 1.65 | 16 | 1.65 |  |
|  |  |  |  |  |  | 1.52 | 17 |  |  |
|  |  |  |  |  |  | 1.77 | 18 |  |  |
| Vit105 | 88 | 2 | 4 | 7.5×10^-4^ | 11657 | 1.94 | 19 | 1.77 |  |
|  |  |  |  |  |  | 1.80 | 20 |  |  |
|  |  |  |  |  |  | 1.57 | 21 |  |  |
| Vit105 | 88 | 2 | 4 | 1×10^-3^ | 11657 | 1.67 | 22 | 1.37 |  |
|  |  |  |  |  |  | 1.11 | 23 |  |  |
|  |  |  |  |  |  | 1.33 | 24 |  |  |
| Vit105 | 88 | 2 | 4 | 1.5×10^-3^ | 11657 | 1.78 | 25 | 1.47 |  |
|  |  |  |  |  |  | 1.52 | 26 |  |  |
|  |  |  |  |  |  | 1.10 | 27 |  |  |
| Vit105 | 88 | 2 | 4 | 2.5×10^-3^ | 11657 | 1.43 | 28 | 1.33 |  |
|  |  |  |  |  |  | 1.19 | 29 |  |  |
|  |  |  |  |  |  | 1.37 | 30 |  |  |
| Vit105 | 88 | 2 | 4 | 5×10^-3^ | 11657 | 1.67 | 31 | 1.64 |  |
|  |  |  |  |  |  | 1.37 | 32 |  |  |
|  |  |  |  |  |  | 1.89 | 33 |  |  |
| Vit105 | 88 | 2 | 4 | 1×10^-2^ | 11657 | 1.99 | 34 | 1.57 |  |
|  |  |  |  |  |  | 1.24 | 35 |  |  |
|  |  |  |  |  |  | 1.47 | 36 |  |  |
| Vit105 | 88 | 1.5 | 3 | 5×10^-4^ | 17732 | 1.25 | 37 | 1.17 |  |
|  |  |  |  |  |  | 1.09 | 38 |  |  |
|  |  |  |  |  |  | 1.18 | 39 |  |  |
| Vit105 | 88 | 2.5 | 5 | 5×10^-4^ | 8413.4 | 1.85 | 40 | 1.82 |  |
|  |  |  |  |  |  | 1.70 | 41 |  |  |
|  |  |  |  |  |  | 1.92 | 42 |  |  |
| Vit105 | 88 | 3 | 6 | 5×10^-4^ | 6348.1 | 1.62 | 43 | 1.65 |  |
|  |  |  |  |  |  | 1.58 | 44 |  |  |
|  |  |  |  |  |  | 1.75 | 45 |  |  |
| Zr_70_Ni_16_Cu_6_Al_8_ | 68 | 2 | 4 | 5×10^-4^ | 10085 | 1.08 | 46 | 1.13 |  |
|  |  |  |  |  |  | 1.18 | 47 |  |  |
|  |  |  |  |  |  | 1.13 | 48 |  |  |
| Zr_70_Ni_16_Cu_6_Al_8_ | 68 | 2.5 | 5 | 5×10^-4^ | 7376.5 | 1.39 | 49 | 1.42 |  |
|  |  |  |  |  |  | 1.44 | 50 |  |  |
|  |  |  |  |  |  | 1.43 | 51 |  |  |
| Zr_65_Cu_15_Ni_10_Al_10_ | 82 | 2 | 4 | 5×10^-4^ | 11222 | 1.91 | 52 | 1.63 |  |
|  |  |  |  |  |  | 1.53 | 53 |  |  |
|  |  |  |  |  |  | 1.44 | 54 |  |  |
| Zr_65_Cu_15_Ni_10_Al_10_ | 82 | 2.5 | 5 | 5×10^-4^ | 8128.9 | 1.66 | 55 | 1.75 |  |
|  |  |  |  |  |  | 1.85 | 56 |  |  |
|  |  |  |  |  |  | 1.73 | 57 |  |  |
| Zr_56_Co_28_Al_16_ | 92 | 2.5 | 5 | 5×10^-4^ | 8520.9 | 1.25 | 58 | 1.24 |  |
|  |  |  |  |  |  | 1.20 | 59 |  |  |
|  |  |  |  |  |  | 1.27 | 60 |  |  |
| Zr_56_Co_28_Al_16_ | 92 | 3 | 6 | 5×10^-4^ | 6412.1 | 1.66 | 61 | 1.63 |  |
|  |  |  |  |  |  | 1.64 | 62 |  |  |
|  |  |  |  |  |  | 1.59 | 63 |  |  |
| Zr_59_Cu_20_Al_10_Ni_8_Ti_3_ | 84.5 | 2 | 4 | 5×10^-4^ | 11564 | 1.42 | 64 | 1.28 |  |
|  |  |  |  |  |  | 1.07 | 65 |  |  |
|  |  |  |  |  |  | 1.36 | 66 |  |  |
| Cu_60_Zr_20_Hf_10_Ti_10_ | 131 | 2 | 4 | 5×10^-4^ | 14112 | 1.41 | 67 | 1.54 |  |
|  |  |  |  |  |  | 1.66 | 68 |  |  |
|  |  |  |  |  |  | 1.54 | 69 |  |  |
| Ti_45_Cu_40_Ni_7.5_Zr_5_  Sn_2.5_ | 110 | 2 | 4 | 5×10^-4^ | 13039 | 1.31 | 70 | 1.22 |  |
|  |  |  |  |  |  | 1.24 | 71 |  |  |
|  |  |  |  |  |  | 1.10 | 72 |  |  |
| *Instron 5869*, $\kappa_{M}=9.47\times10^{7}$N.m^-1^ | | | | | | | | | |
| Vit105 | 88 | 3 | 6 | 2.5×10^-4^ | 6999.8 | 1.90 | 73 | 1.79 |  |
|  |  |  |  |  |  | 1.70 | 74 |  |  |
|  |  |  |  |  |  | 1.78 | 75 |  |  |
| Vit105 | 88 | 2 | 4 | 5×10^-4^ | 12715 | 1.61 | 76 | 1.51 |  |
|  |  |  |  |  |  | 1.47 | 77 |  |  |
|  |  |  |  |  |  | 1.44 | 78 |  |  |
| *Instron 3384*, $\kappa_{M}=1.34\times10^{8}$ N.m^-1^ | | | | | | | | | |
| Vit105 | 88 | 3 | 6 | 5×10^-4^ | 8280.2 | 1.36 | 79 | 1.33 |  |
|  |  |  |  |  |  | 1.14 | 80 |  |  |
|  |  |  |  |  |  | 1.48 | 81 |  |  |
| Zr_65_Cu_15_Ni_10_Al_10_ | 82 | 2.5 | 5 | 5×10^-4^ | 10257 | 1.47 | 82 | 1.58 |  |
|  |  |  |  |  |  | 1.59 | 83 |  |  |
|  |  |  |  |  |  | 1.67 | 84 |  |  |
| Zr_65_Cu_15_Ni_10_Al_10_ | 82 | 3 | 6 | 5×10^-4^ | 7951.7 | 1.89 | 85 | 1.89 |  |
|  |  |  |  |  |  | 1.87 | 86 |  |  |
|  |  |  |  |  |  | 1.91 | 87 |  |  |
| Zr_62_Cu_15.5_Ni_12.5_Al_10_ | 82 | 2 | 4 | 5×10^-4^ | 13628 | 1.08 | 88 | 1.10 |  |
|  |  |  |  |  |  | 1.12 | 89 |  |  |
|  |  |  |  |  |  | 1.09 | 90 |  |  |

**Text S1. Calculation of the shear offset of shear band during a serrated shear event**

During a serrated event, the sliding of shear band will cause the release of the elastic energy and the stress drop in the stress-time/strain curves. Assuming that the shear band sliding causes a vertical displacement $\Delta x$ along the compression direction in a serrated event, according to **Eq.1** in the main text, we have:

$-\Delta\sigma_{A}=k(v_{0}\Delta t_{s}-\Delta x)$ (1)

where $v_{0}$ is the external loading rate, $k$is the elastic constant of the machine-sample system, $\Delta t_{s}$ and $\Delta\sigma_{A}$ is the duration of the serrated event and the stress drop during the event, respectively. From **Eq. S1**, we could immediately obtain the $\Delta x$:

$\Delta x=v_{0}\Delta t_{s}+\Delta\sigma_{A}/k$ (2)

From the geometrical relations between the shear offset and the vertical displacement, $\Delta x=\delta\cos\theta$ where $\theta$ is the angle of the shear band with the loading axis, one can obtain the shear offset during a serrated event:

$\delta=(v_{0}\Delta t_{s}+\Delta\sigma_{A}/k)/\cos\theta$ (3)

Thus, if the duration of serrated event $\Delta t_{s}$ and the stress drop $\Delta\sigma_{A}$ during the event can be precisely measured, one could calculate the shear offset of shear band in a serrated event according to **Eq. S3**.

**Text S2. Estimation of the temperature rise $\Delta T$ during a shear band sliding event**

Once activated, the shear band can be either cooperatively sliding on the primary shear plane or progressively proceeds with a shear front. Here, we use the former shear mode, which has been verified by many experimental studies^1-3^. The temperature rise calculation mainly follows the method of Ref.[2]. Specifically, we consider the shear band as a two-dimensional heat source with the heat flux $f(t)$ and the sample body as a three-dimensional heat conducting medium. As the energy source for the temperature rise is mainly from the plastic work of the shear band, the heat flux $f(t)$ should be proportional to plastic energy rate or the shear-band sliding velocity. For a first-order approximation, we have:

$f(t)=\sigma_{A}v\sin\theta$ (4)

Where $v$ is the instantaneous SBV at time $t$, the flow stress $\sigma_{f}$ in the band is roughly taken as the applied stress $\sigma_{A}$ for a first-order approximation. According to the heat conduction theory^4^, the instantaneous temperature rise $\Delta T$ is calculated by:

$\Delta T=\frac{1}{2\rho c_{p}\sqrt{\pi a}}\int_{0}^{t} f(t-\tau)\frac{d\tau}{\sqrt{\tau}}$ (5)

where $\rho$ is the sample mass density, $c_{p}$and $\alpha$ are the heat capacity and the thermal diffusivity of the MG material, respectively. As the stress and SBV profile can be experimentally resolved in a shear-banding event in our current work, the instantaneous temperature rise can be numerically integrated from **Eq.S5**. A typical example for the numerical calculation of $\Delta T$ in the serrated event corresponding to the CSBV for the sample (Vit105, $D=2$ mm, s^-1^, $\kappa_{M}=7.792\times10^{7}$ N.m^-1^) is shown in **Fig.S5.** The parameter values used in the integration: $\rho=6.694\times10^{3}$ kg/m^3^, $c_{p}=350$ J/(kg. K), $\alpha=2.2\times10^{-6}$m^2^/s, $\theta=45^{\circ}$ taken from Refs. [2] and [5]. As can be seen, the maximum temperature rise, $\Delta T_{max}<5$ K. Similar results are also obtained for other MG samples. Therefore, it is reasonable to treat the shear band sliding as an isothermal process.

**Text S3. Numerical calculation of the characteristic SBV, $v_{m}$, with the parameter of $k/k_{cr}$ as a function of various intrinsic and extrinsic factors.**

We used the stick-slip dynamic equations in Ref. [6] to describe the shear band sliding process. The constitutive flow law of the band is given by the cooperative shear model (CSM)^7^ and the evolution of the internal state in the band is described by the effective disorder temperature dynamics^8^. For numerical calculations, these equations are simply listed as follows:

(6.1)

(6.2)

$\sigma_{b}(v,x)=\sigma_{b0}\left\{1-(k_{B}T/W_{0})^{2/3}[\ln(v_{s}/v)-1/\chi]^{2/3}\right\}$ (6.3)

where $\sigma_{b}$ is the flow stress in the shear band, $\hat{\chi}(v)=\chi_{w}/\ln(v_{c}/v)$ is the steady state of the effective disorder temperature $\chi$at the band velocity $v$. Other parameters are constants and their definitions can be found in Ref. 6. By performing the linear stability analysis, we have showed that the appearance of stick-slip shear-band sliding or serrated flow behavior is controlled by the parameter $k/k_{cr}$^6^: for $k/k_{cr}<1$, stick-slip shear-band sliding appears, otherwise the shear band slides at the constant loading rate $v_{0}$. $k_{cr}$ is a function of the loading rate $v_{0}$ and the testing temperature $T$:

$k_{cr}=\alpha\sigma_{b0}(k_{B}T/W_{0})^{2/3}\ln(v_{c}/v_{0})/C_{0}$ (7)

where $\alpha$ and $C_{0}$ are constants, with $G_{0T}$ the shear modulus at 0 K and other parameters constants. While $k$ is a general function of the Young’s modulus $E$, the sample size (the diameter $D$ and the length $L$) and the machine stiffness $\kappa_{M}$: $k=k(E,D,L,\kappa_{M})$, and for a cylinder sample in the uniaxial compression, $k=E/[L+\pi D^{2}E/(4\kappa_{M})]$. Thus, the stick-slip dynamics of shear band is influenced by various intrinsic and extrinsic factors, which can be reflected from $k/k_{cr}$.

We numerically solved **Eq.S6** at different values of $k/k_{cr}$ (0.45, 0.5, 0.6, 0.7, 0.8, 0.9, 0.95). The parameters used in the calculation are: $\sigma_{b0}=1$ GPa, $\chi{}_{w}=0.5$, $c_{0}=10^{-5}$ m.s^-1^, $v_{s}=10^{2}$ m.s^-1^, $v_{c}=1$ m.s^-1^, $(k_{B}T/W)^{2/3}=0.05$ and the loading rate $v_{0}=10^{-8}$ m.s^-1^. These parameters give $k_{cr}=1951$ GPa.m^-1^ according to **Eq. S7**. To accelerate the calculation process, we used an adaptive time-step Euler method and neglected the effect of inertia ($m=0$). The initial conditions for the calculation are the steady-state solutions of **Eq. S6**: $\chi(t=0)=\hat{\chi}(v_{0})=0.05157$, $v=v_{0}=10^{-8}$m.s^-1^, $\sigma(t=0)=\sigma_{b}(t=0)=0.88176$GPa.

The calculated stress-time curves at different $k/k_{cr}$ is shown in **Fig.S6**, where the shear band is initially sliding a steady velocity (the loading rate) and then some perturbations gradually developed into stable stress serrations. The shear band stick-slip motions are repeated during these stable serrations. For each case, the characteristic shear band sliding velocity, $v_{m}$, (the maximum velocity that the band reaches in a serrated event) is also extracted [see **Fig.S6 (e) and (f)**] and also plotted against $k/k_{cr}$, as displayed in **Fig.5 (c)**. One can see that with the increase of $k/k_{cr}$, $v_{m}$ is gradually decreased and far away from the CSBV, thus in general indicating a increased ductility in MGs.

**
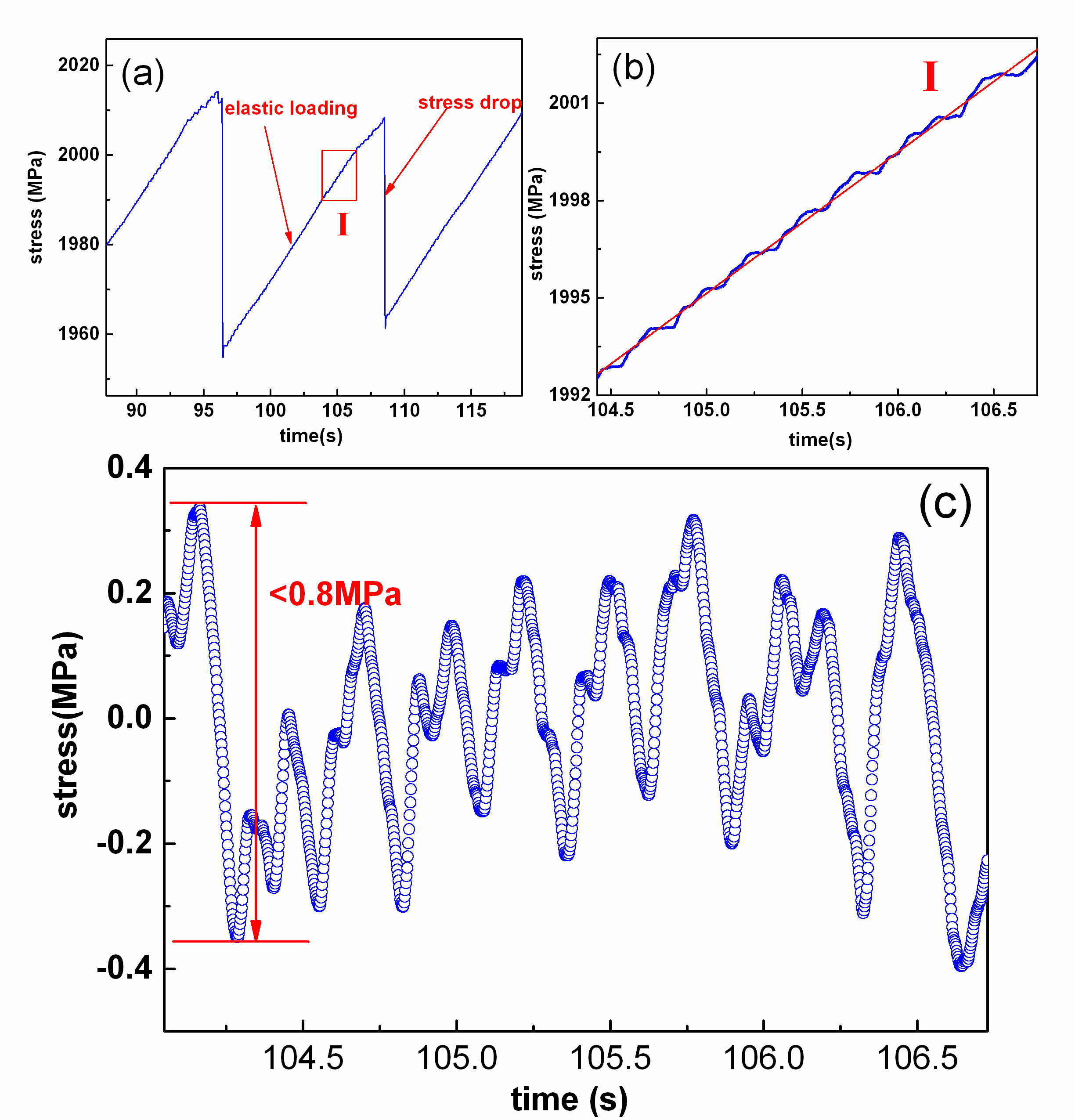
**

**Figure S1**. (a) A segment of stress-time curve recorded for a Vit105 sample (strain rate 1×10^-4^ s^-1^, Instron 5567), showing the serrations. A serration is composed of the elastic loading part and the stress drop part. (b) The enlarged view of region I showing obvious stress noises in the elastic loading part. (d) The stress noise level (<0.8 MPa) obtained by subtracting the linear fitting part from the stress-time curve.


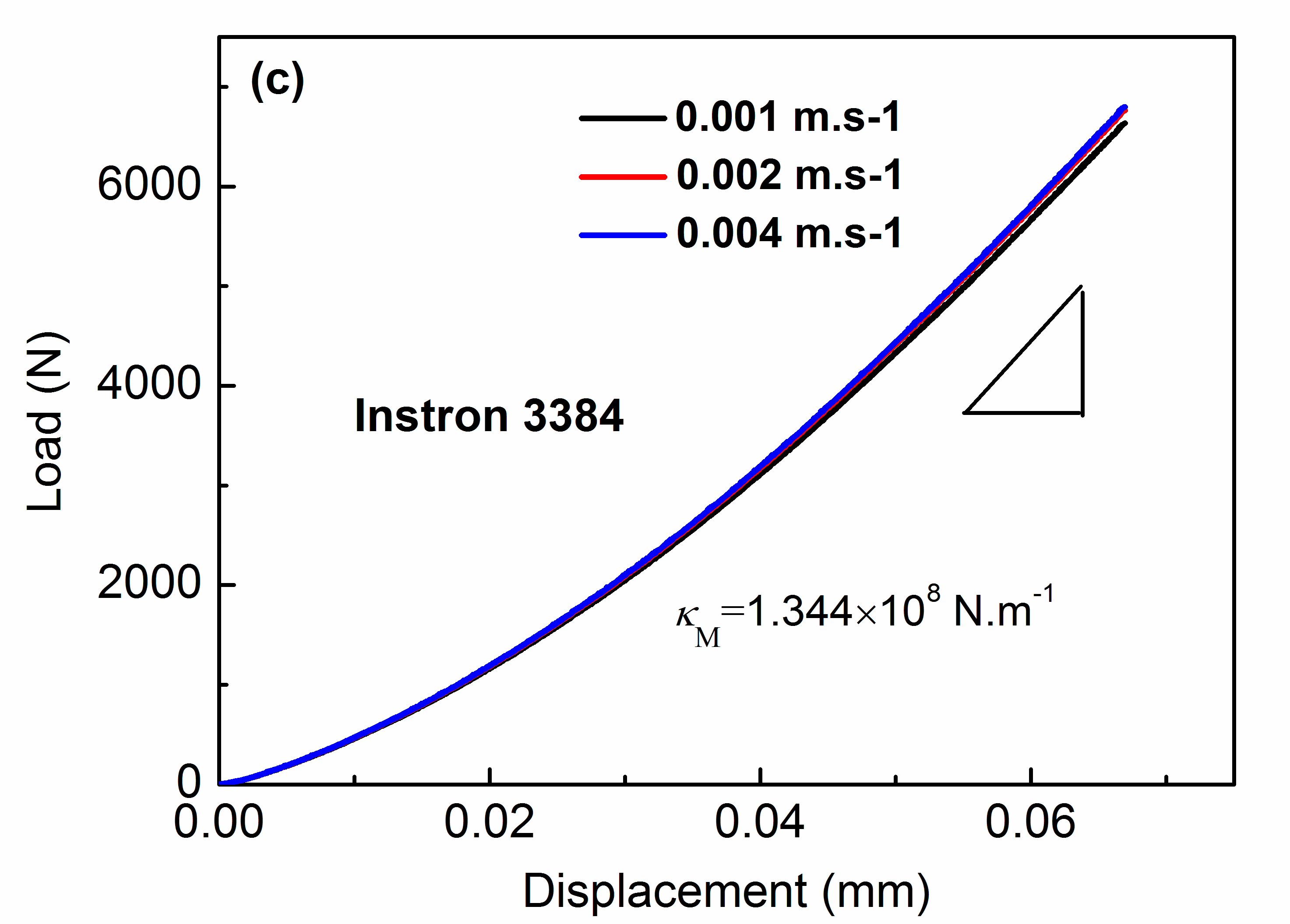

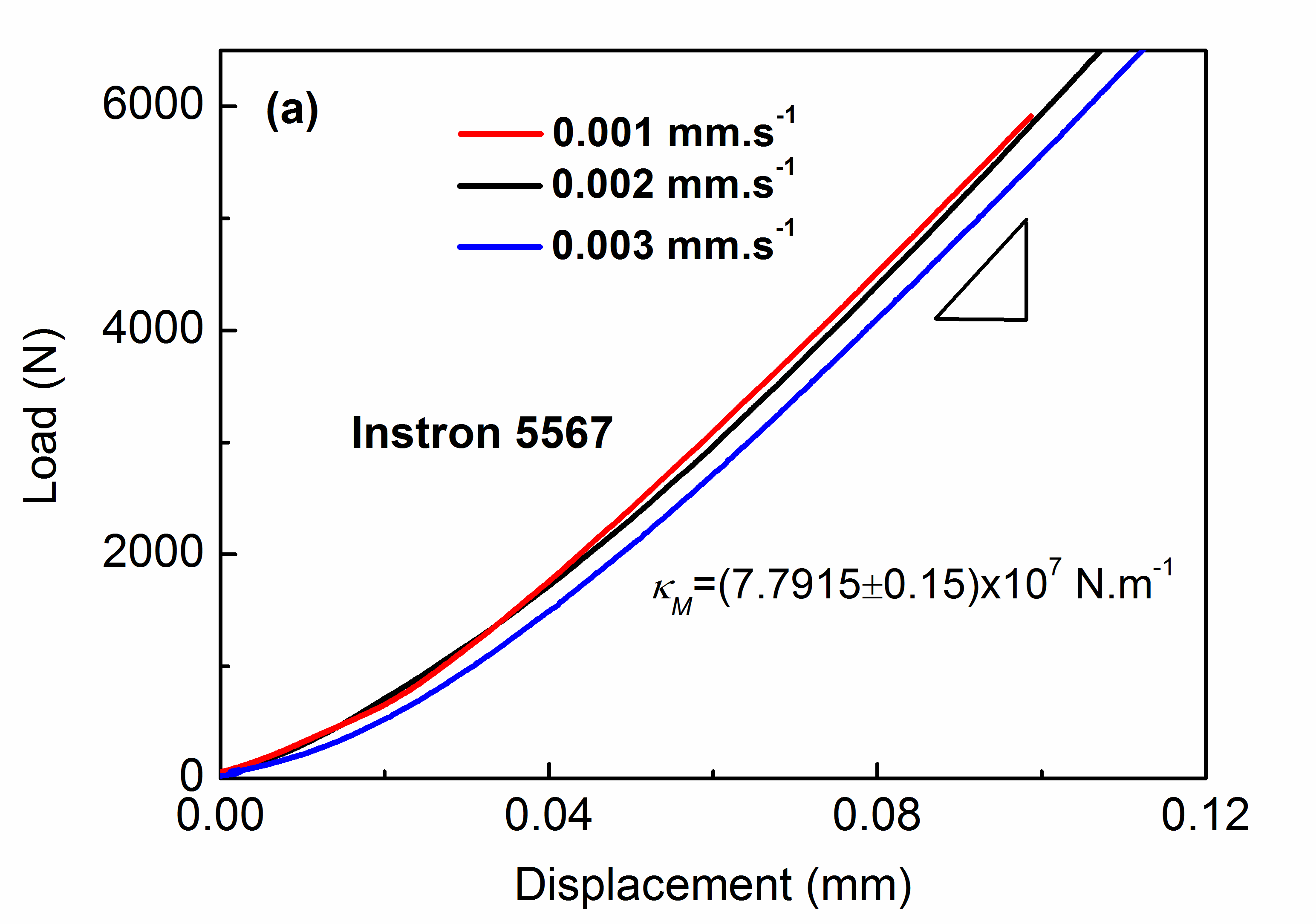

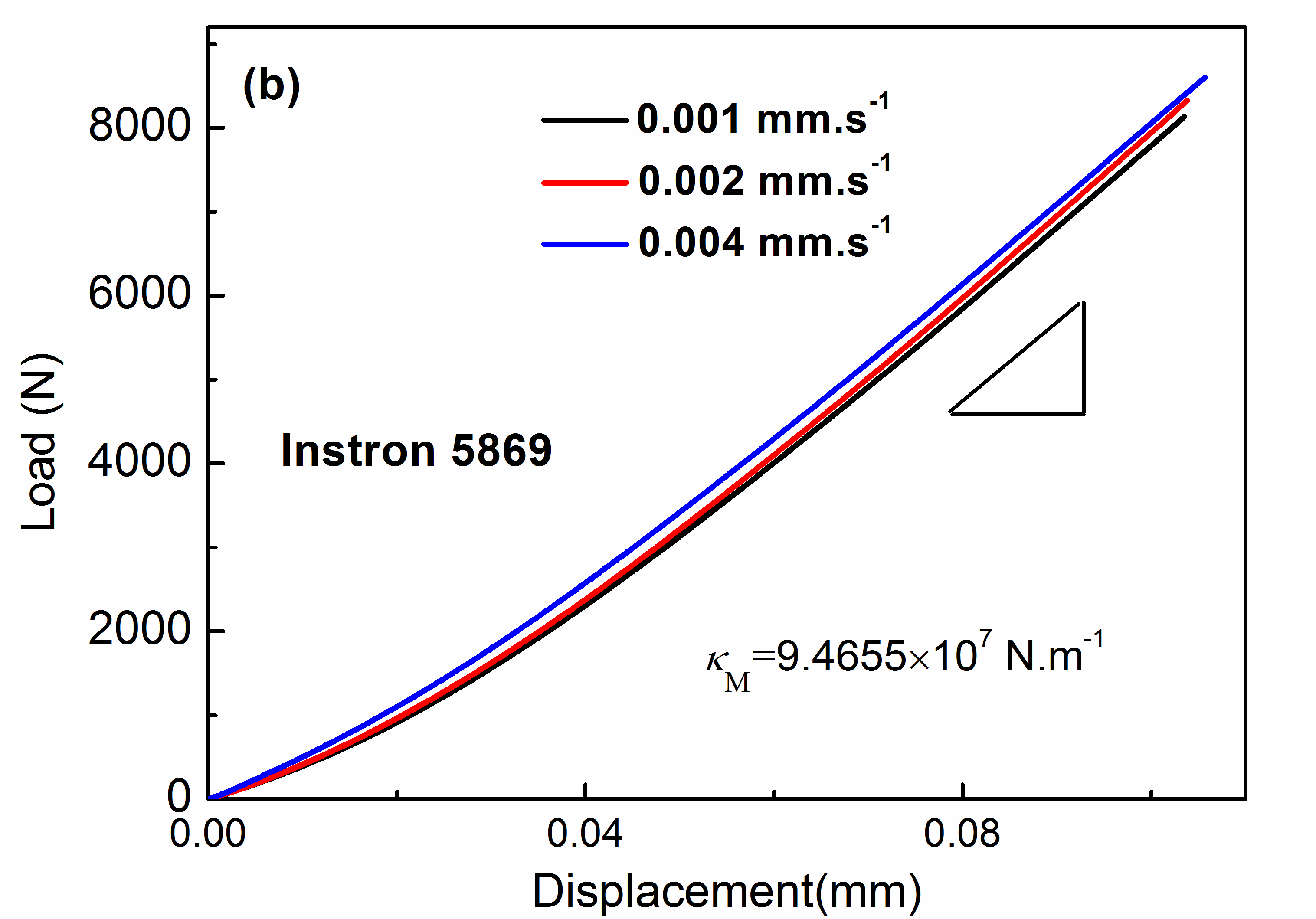


**Figure S2.** The load-displacement curves of the testing machine Instron 5567 (a), Instron 5869 (b) and Instron 3384 (c) loaded at the rate of 0.001, 0.002 and 0.004 mm.s-1, respectively, from which the machine stiffness can be determined (the slope of the curve).


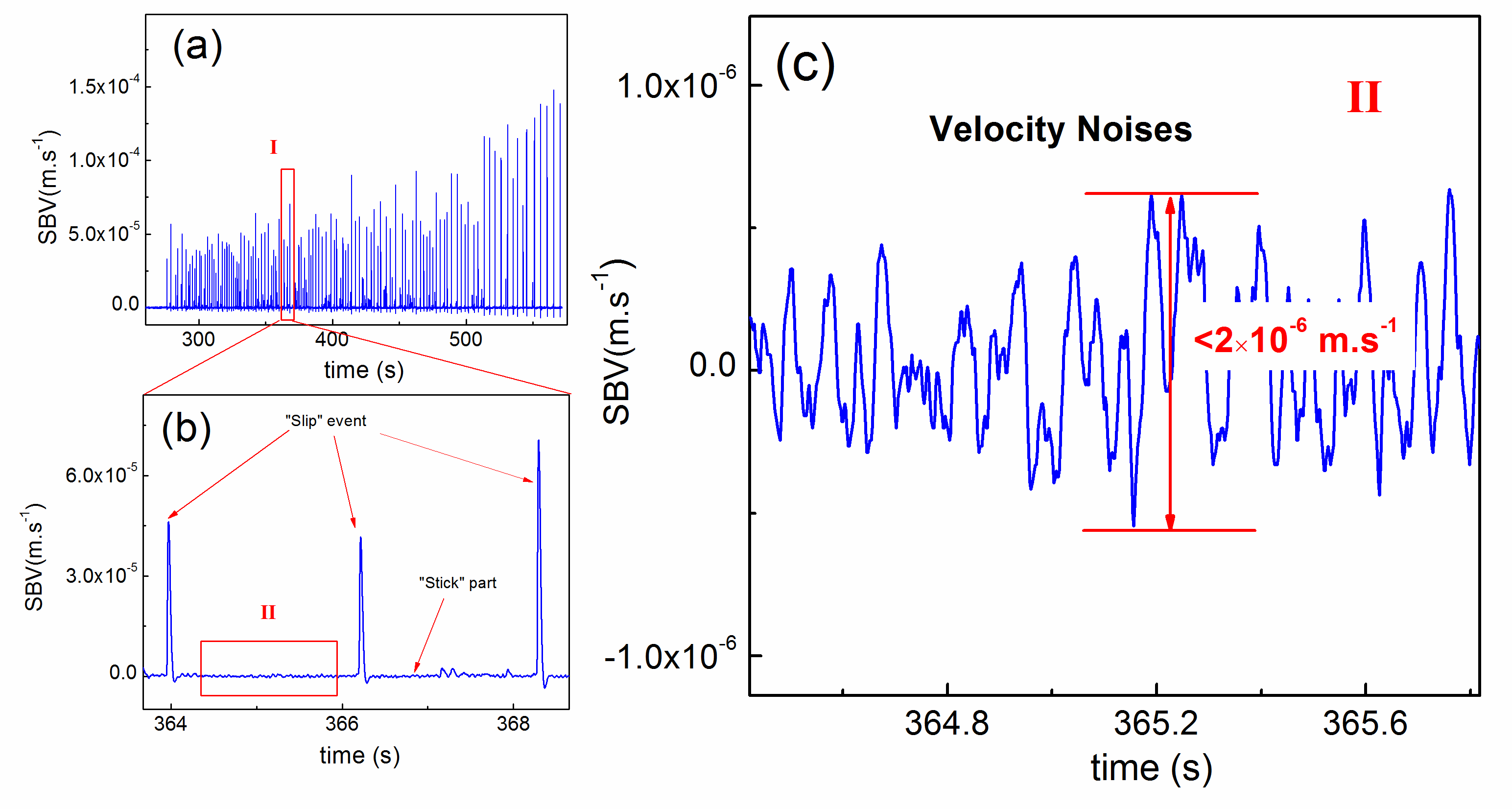


**Figure S3.** The determination of the noise level in the SBV profile: **(a)** the typical SBV profile for the sample (Vit105, $D=2$ mm, s^-1^ under Instron 5567). **(b)** The enlarged view of region I showing the “slip” event and the “stick” part in the profile. **(c)** The enlarged view of region II showing that the noises in the “stick” part are less than $2.0\times10^{-6}$ m.s^-1^.


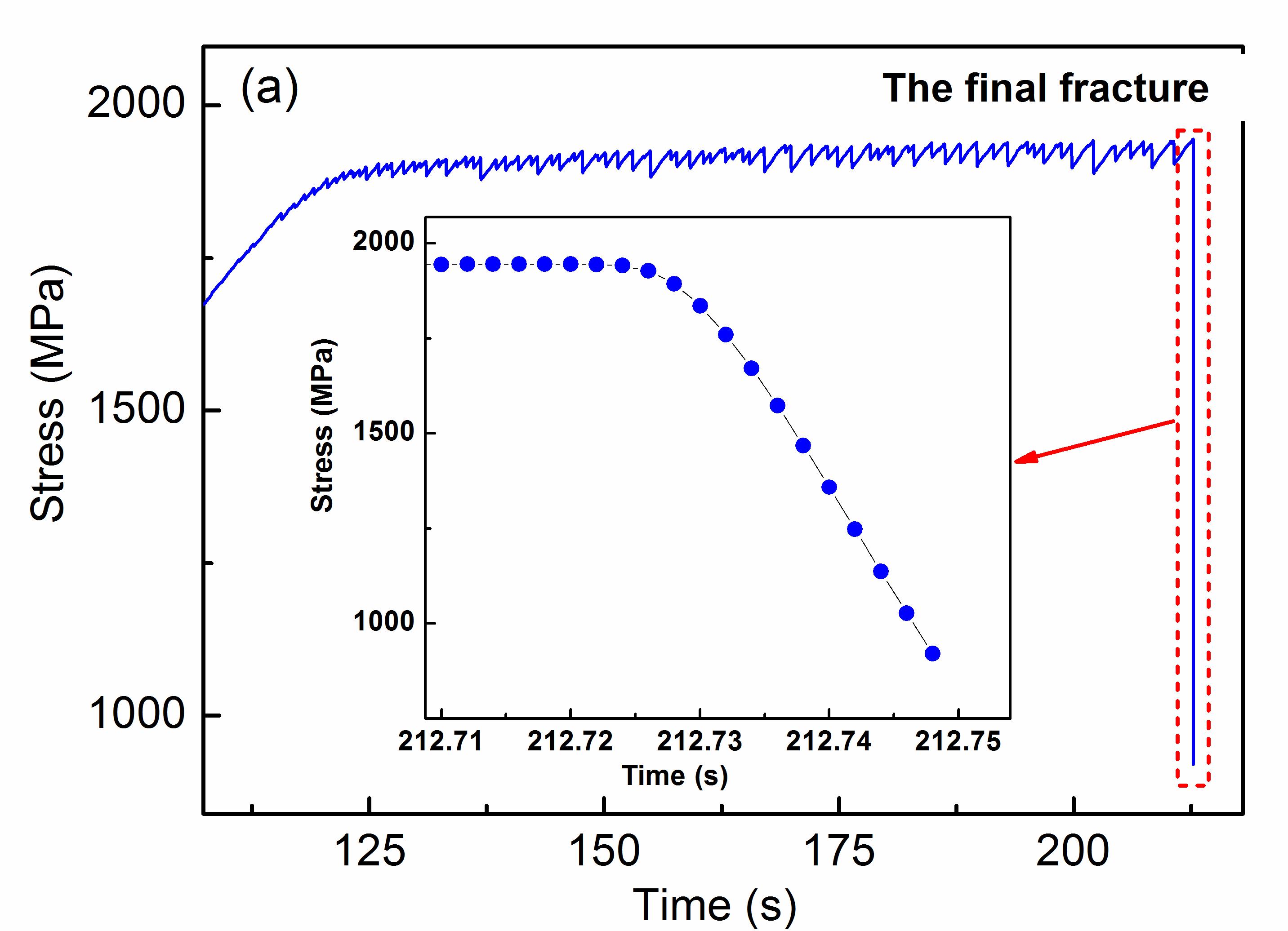


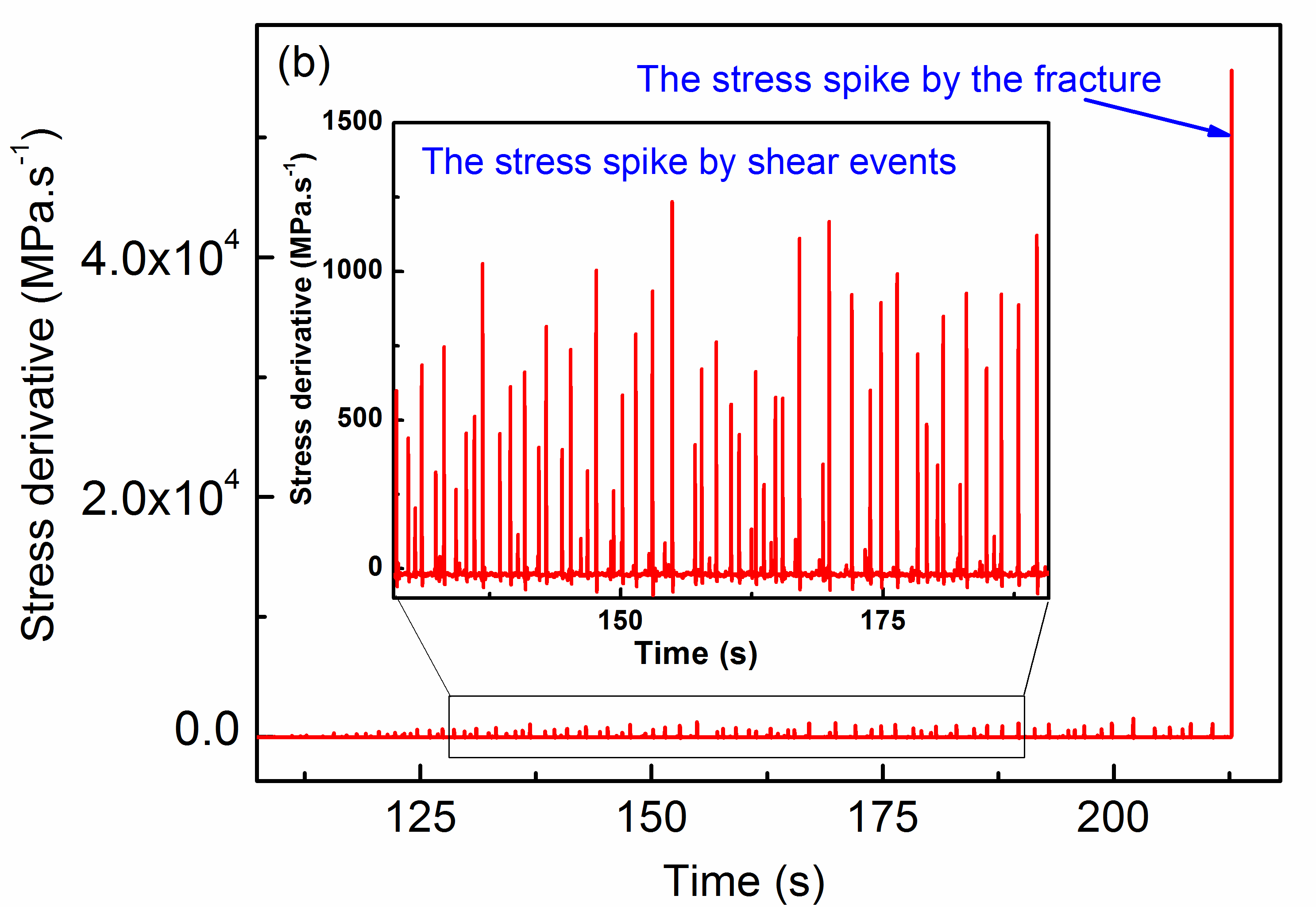


**Figure S4. (a)** The stress-time curve for a typical sample (Vit105, *D*=2.5 mm at the strain rate 5×10^-4^ s^-1^), displaying the stress-time data points at the final fracture process. **(b)** The stress derivative corresponding to (a), where the stress spike induced by the fracture is much larger than that of serrated events.


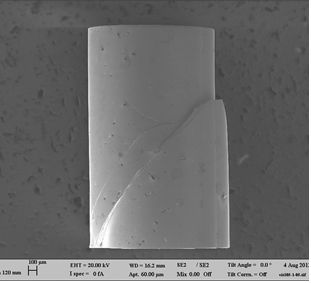

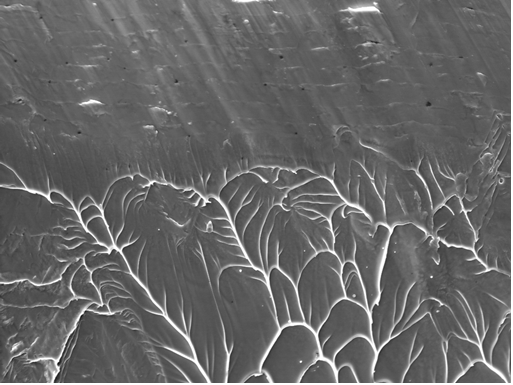


**1 mm**

**(a)**

**Shear direction**

**stick-slip region**

**catastrophic failure region**

**Vein-like patterns**

**Primary shear band**

**(b)**

**25 um**

**Figure S5. (a)** The SEM side view of a MG sample (Vit105, $D=1.5$mm, s^-1^ under Instron 5567 after deformation before the final fracture, where a primary shear band can be seen. **(b)** The fracture surface morphology along the primary shear band after the final fracture, where the micrometer-scale stripes due to the stick-slip motion of shear band and the vein-like patterns due to the catastrophic failure can be seen.


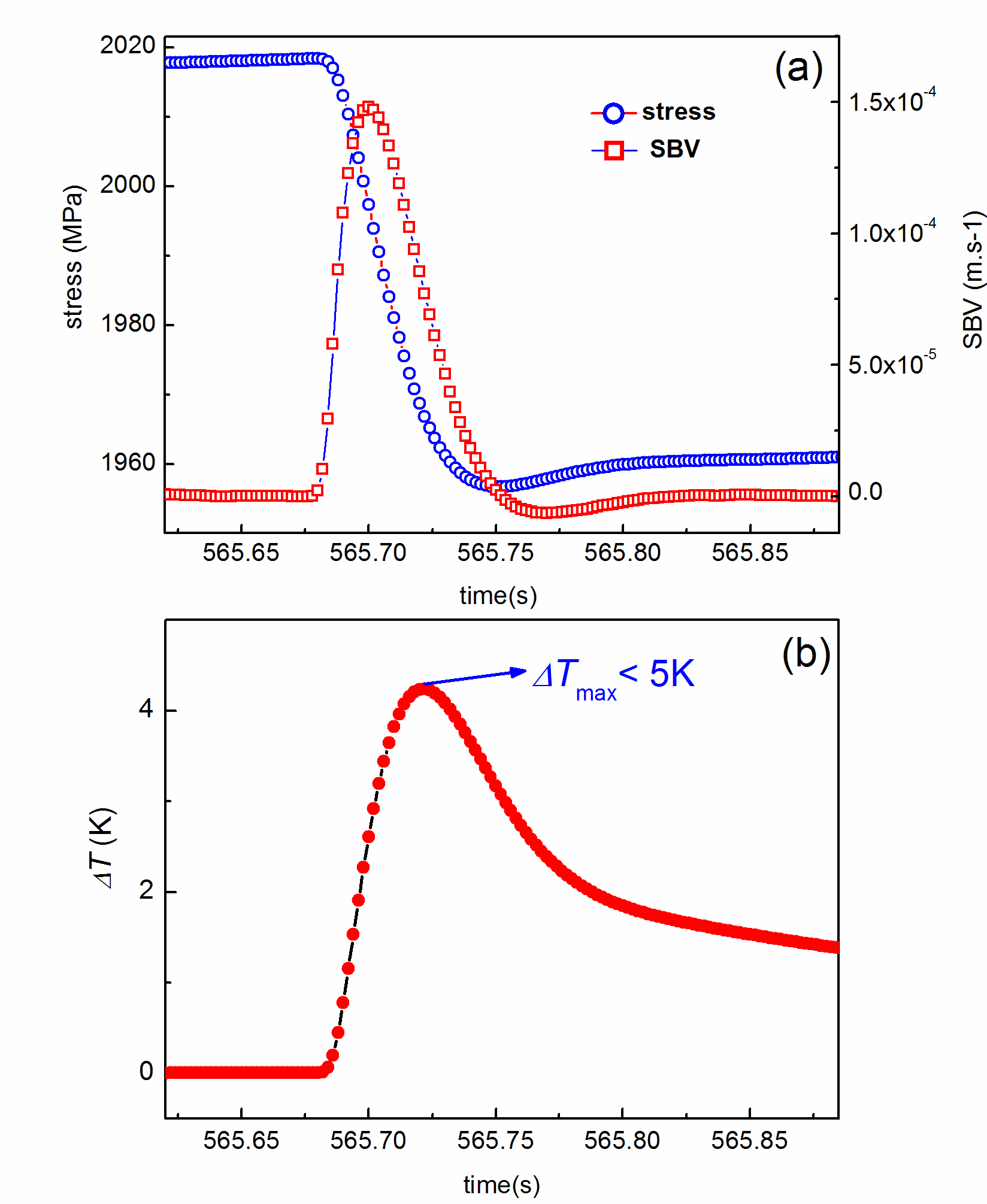


**Figure S6.** The numerical integration of the temperature rise for a typical sample (Vit105, $D=2$mm, s^-1^, $\kappa_{M}=7.792\times10^{7}$N.m^-1^): **(a)** The stress and shear-band velocity profile in the serrated event corresponding to the CSBV. **(b)** The calculated instantaneous temperature rise curve corresponding to the curves in (a), where one can see that the maximum temperature rise is less than 5 K. The parameter values used in the integration: $\rho=6.694\times10^{3}$ kg/m^3^, $c_{p}=350$J/(kg. K), $\alpha=2.2\times10^{-6}$ m^2^/s , $\theta=45^{\circ}$, taken from Refs. 2 and 4.


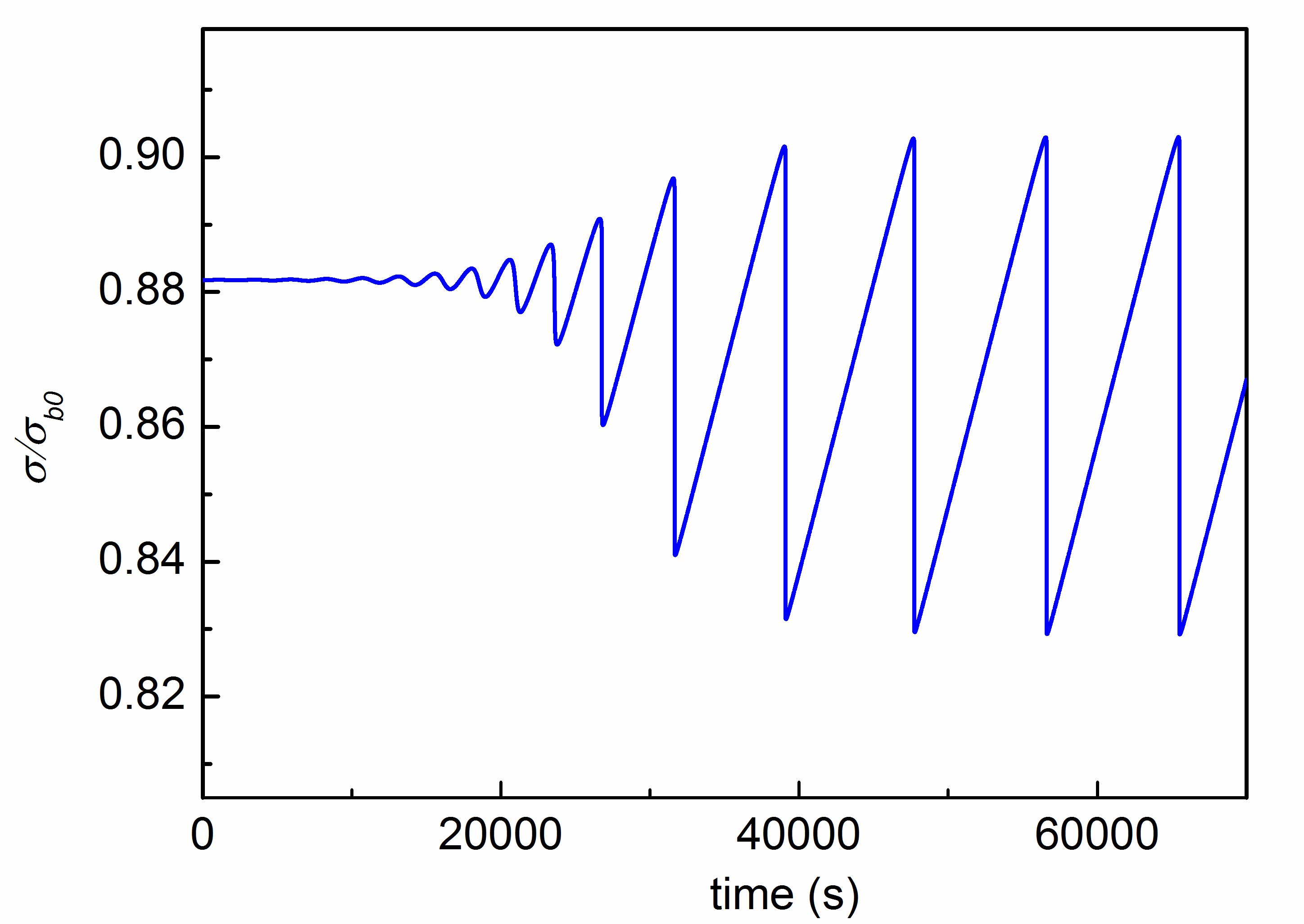

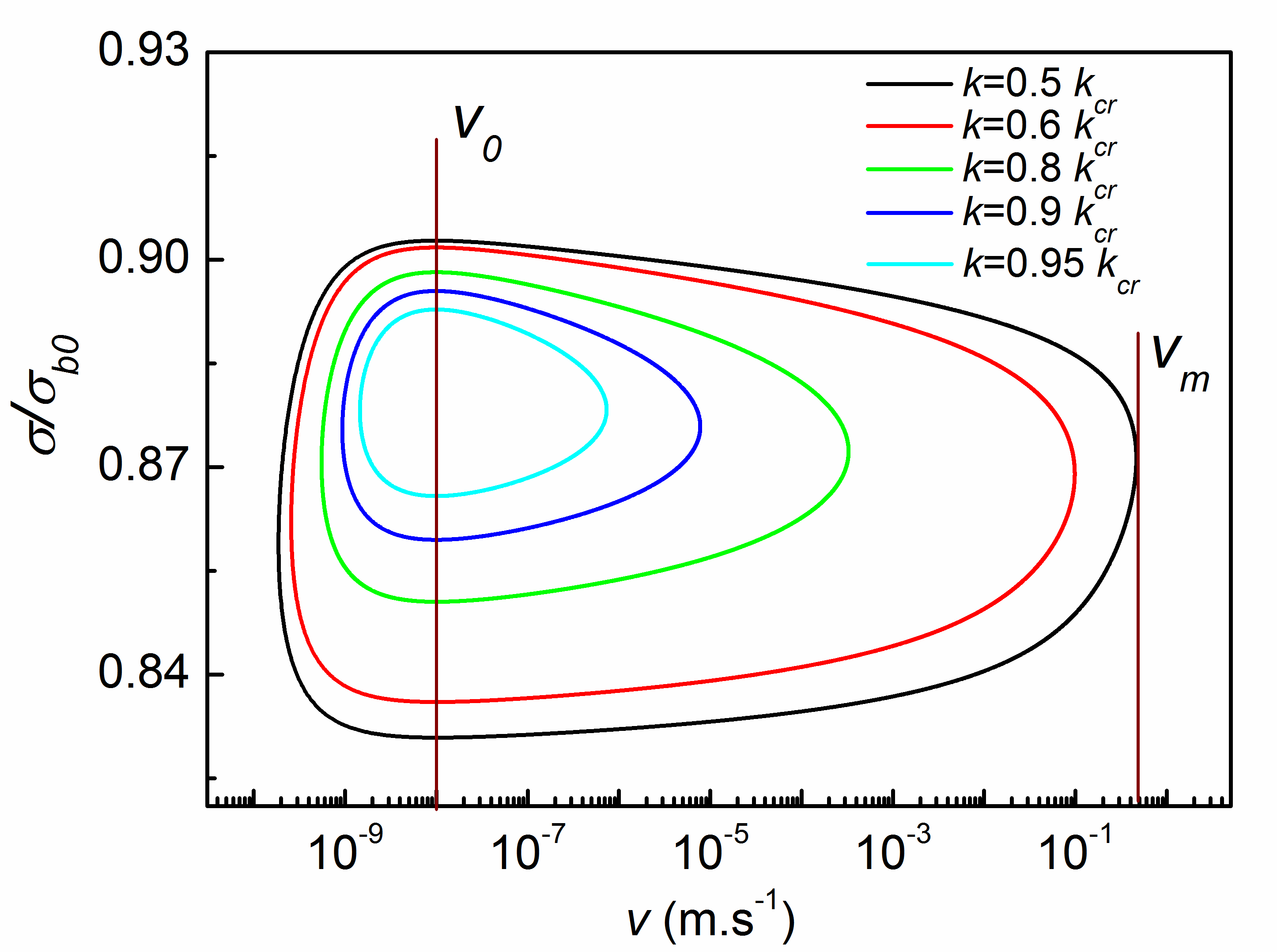

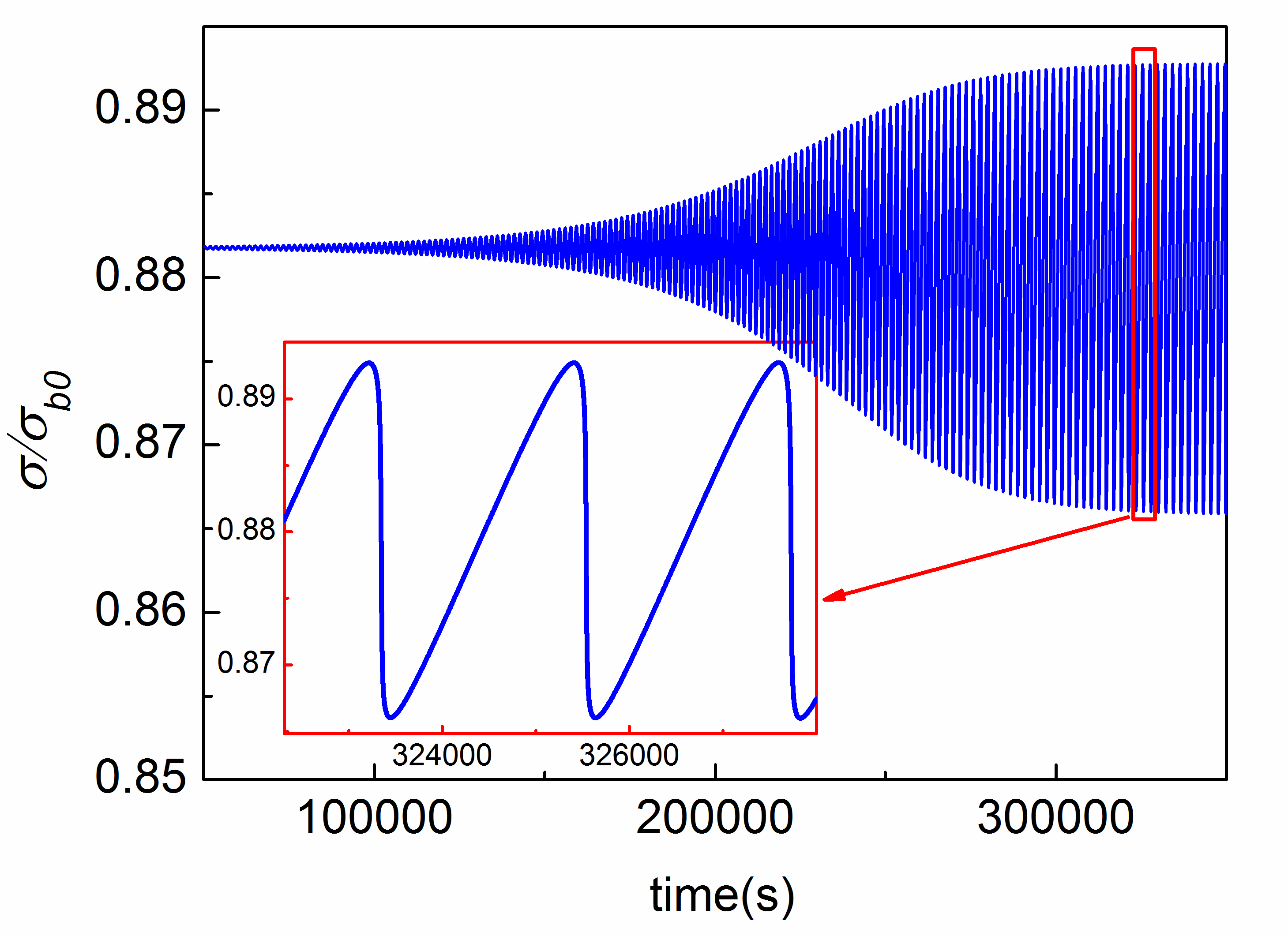

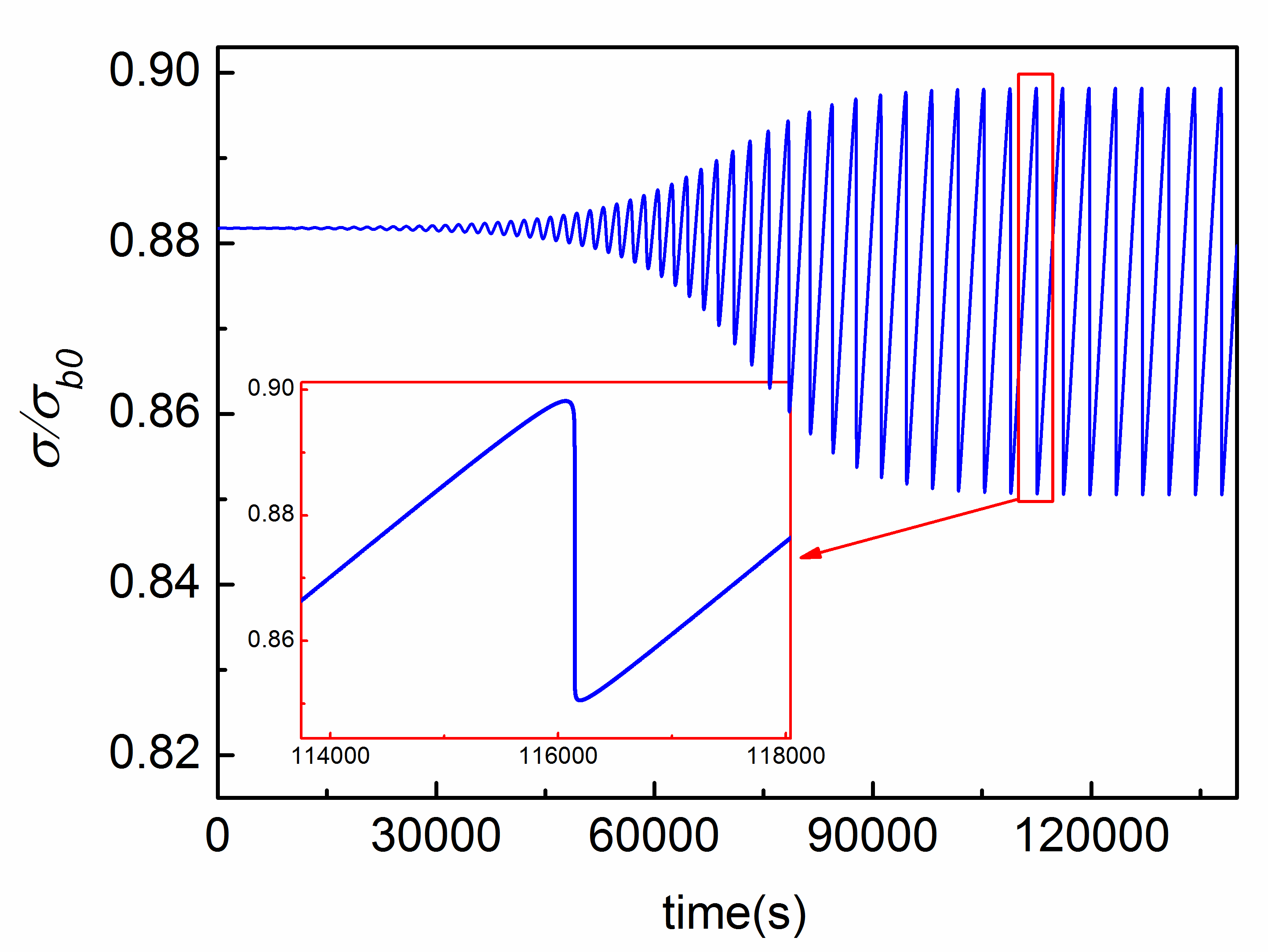

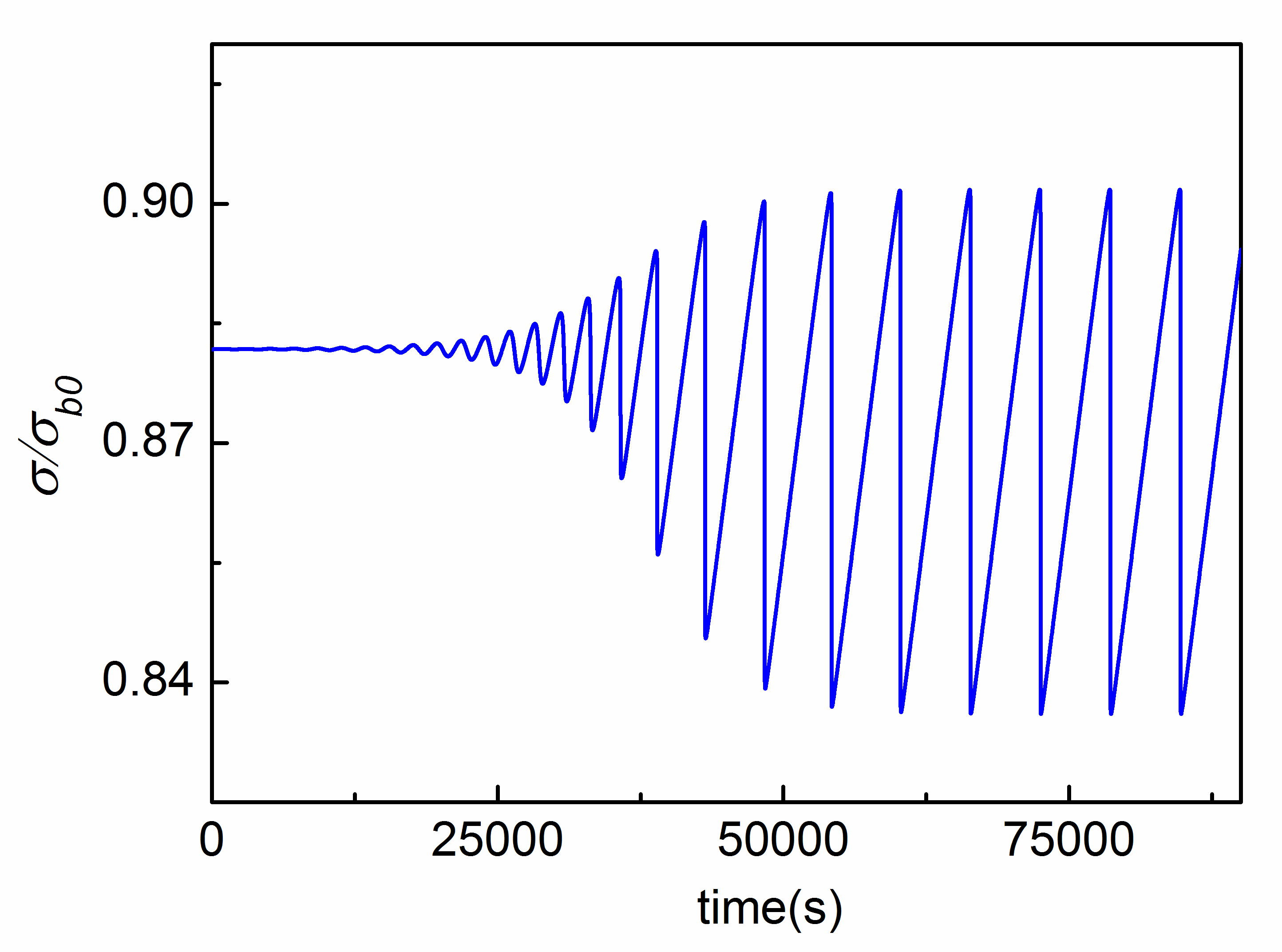

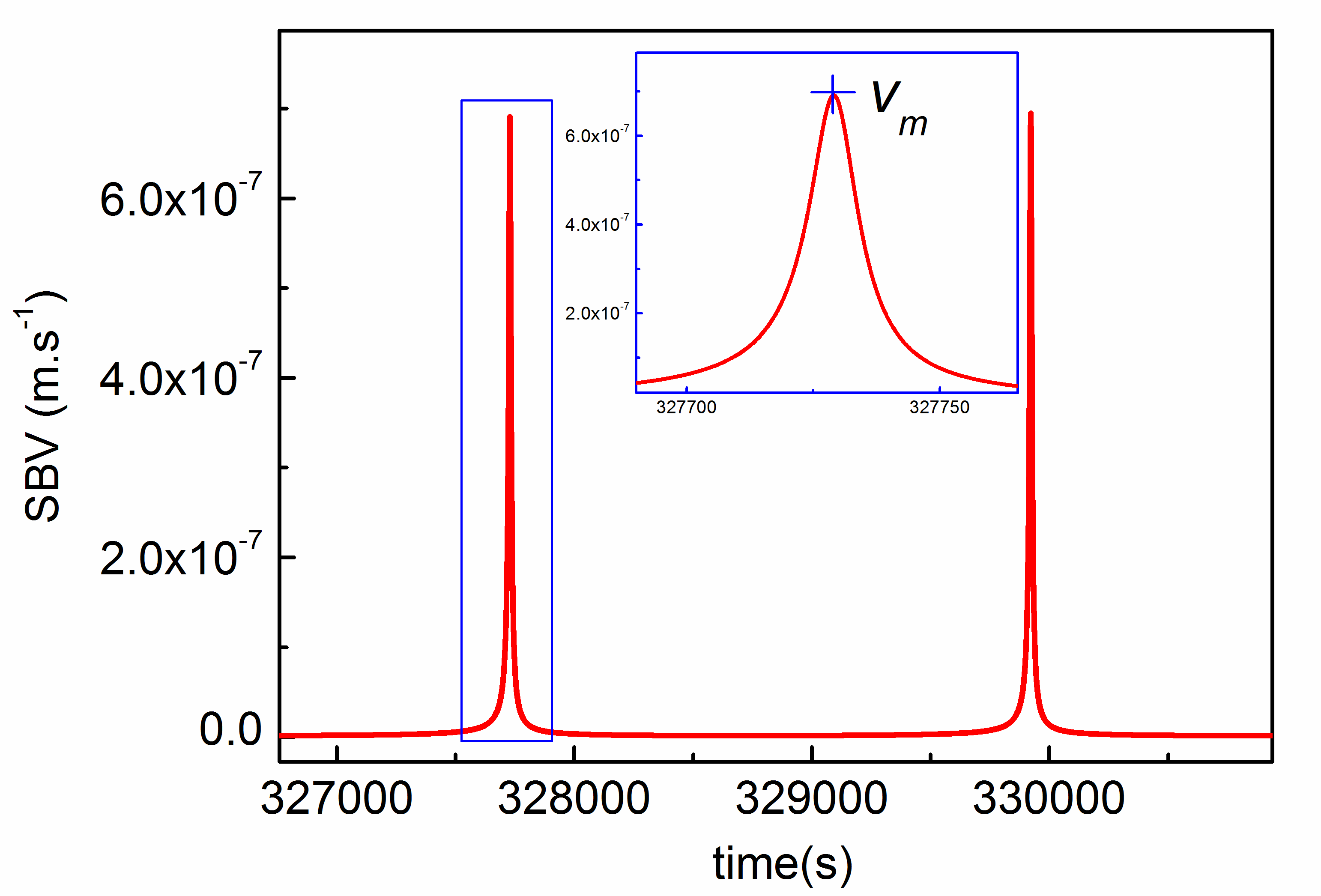


*k* =0.45 *k_cr_*

(b)

(c)

*k* =0.6 *k_cr_*

*k* =0.8 *k_cr_*

*k* =0.95 *k_cr_*

(d)

(e)

(f)

**Figure S7.** Numerical calculation results of a single shear band dynamics. **(a)-(d)** The stress-time curves at $k=0.45k_{cr}$, $0.6k_{cr}$, $0.8k_{cr}$, $0.95k_{cr}$, respectively. One can see that periodic stable serrations can be developed for each case. **(e)** The calculated SBV profile showing the repeated stick-slip motions of the shear band at $k=0.95k_{cr}$, from which the characteristic shear band sliding velocity, $v_{m}$, (the maximum velocity that the band reaches in a serrated event) can be determined. **(f)** The phase space of $\sigma-v$ at stable stick-slip shear banding for different values of $k/k_{cr}$.

**References:**

[1]. Song SX, Bei H, Wadsworth J, Nieh TG. Flow serration in a Zr-based bulk metallic glass in compression at low strain rates. *Intermetallics* **16**, 813-818 (2008).

[2]. Cheng YQ, Han Z, Li Y, Ma E. Cold versus hot shear banding in bulk metallic glass. *Phys Rev B* **80**, 134115 (2009).

[3]. Han Z, Li Y, Cooperative shear and catastrophic fracture of bulk metallic glasses from a shear-band instability perspective. *J Mater Res* **24**, 3620-3627 (2009).

[4]. Carslaw HS, Jaeger JC, Conduction of Heat in Solids. Oxford University Press, Oxford, 1948, p.76.

[5]. Wang WH, The elastic properties, elastic models and elastic perspectives of metallic glasses. *Prog Mater Sci* **57**, 487-656 (2012).

[6]. Sun BA, Pauly S, Hu J, Wang WH, Kühn U, Eckert J. Origin of intermittent plastic flow and instability of shear band sliding in bulk metallic glasses. *Phys Rev Lett* **110**, 225501 (2013).

[7]. Johnson WL, Samwer K, A Universal Criterion for Plastic Yielding of Metallic Glasses with a (T/T_{g})^{2/3} Temperature Dependence. *Phys Rev Lett* **95**, 195501 (2005).

[8]. Langer JS, Manning ML, Steady-state effective temperature dynamics in glassy materials. *Phys Rev E* **76**, 056107 (2007).
